# Inhibition of KDEL Receptors Remodels the Tumor Microenvironment for T Cell Independent Tumor Regression

**DOI:** 10.1002/advs.76148

**Published:** 2026-06-23

**Authors:** Shakti P Pattanayak, Hong Wang, Belinda Willard, Timothy A. Chan, William C Merrick, Zheng‐Rong Lu, Boaz Tirosh

**Affiliations:** ^1^ Department of Biochemistry Case Western Reserve University School of Medicine Cleveland Ohio USA; ^2^ Department of Biomedical Engineering Case Western Reserve University School of Medicine Cleveland Ohio USA; ^3^ Proteomics and Metabolomics Shared Laboratory Resource Lerner Research Institute, Cleveland Clinic Cleveland Ohio USA; ^4^ Center for Immunotherapy and Precision Immuno‐Oncology Cleveland Clinic Cleveland Ohio USA

**Keywords:** calreticulin, cancer immunity cycle, ER stress, immunogenic cell death, innate immunity

## Abstract

Tumor immunotherapy is supported by low‐grade inflammatory conditions in the microenvironment, triggered by immunogenic cell death (ICD). However, ICD is dampened when tumors acquire resistance, affecting immune recognition. KDEL receptors (KDELRs), through a retrograde Golgi‐to‐ER transport, prevent spontaneous secretion of KDEL proteins. We report that inhibition of a single KDELR in a minor fraction of tumor cells, primarily KDELR2, provokes robust infiltration of macrophages and neutrophils into the tumor microenvironment, resulting in regression of both immunogenic and non‐immunogenic tumors initially independently of T cells. Importantly, in the course of regression, anti‐tumor T cells are primed, conferring protection against a second challenge. Recapitulated by intratumoral delivery of siDKELR2 utilizing lipid nanoparticles, we implicate KDELR2 as a target to unleash an unusual robust innate immune response, which represents a tractable approach to initiate an adaptive response downstream, bypassing conventional ICD‐inducing therapies. We propose KDELR targeting as a strategy to improve immunotherapy across tumor types, including “cold” tumors resistant to T cell‐based immunotherapies.

## Graphical abstract

Inhibition of KDELR2 in a small fraction of tumor cells generates sustainable immunogenic cell death conditions within the tumor microenvironment. These conditions promote the regression of tumors in a T cell independent manner. During regression, macrophages prime T cells that subsequently provide systemic protection against recurrence. The potency of the effect makes it amenable for a nanoparticle‐mediated delivery of siKDELR2.

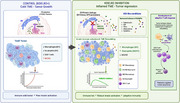

## 1 Introduction

The cancer immunity cycle (CIC) model has been evoked to describe the iterative steps by which the anti‐cancer immune response is perpetuated and adapts to evolving tumors. A productive CIC is required to achieve a durable immune response and is dependent on the maintenance of inflammatory conditions in the tumor microenvironment (TME), which switches the immune response from tolerogenic to immunogenic [1]. Inflammatory conditions in situ facilitate the productive activation of effector immune cells, primarily T cells and natural killer (NK) cells [2], and are therefore highly desired for cancer immunotherapy [3, 4]. A major driver of the inflammatory TME is the immunogenic cell death (ICD) of tumor cells, which is a form of cell death that attracts macrophages and dendritic cells (DCs) into the tumor tissue [5]. ICD is typically generated when cell death is accompanied by cellular stress, most commonly induced by cytotoxic cancer therapies such as chemotherapy [6], radiotherapy [7], and photodynamic therapy [8]. However, this dependency on therapy‐induced stress represents a major limitation. As tumors acquire resistance to these treatments, stress signaling is attenuated, leading to reduced ICD and a gradual loss of inflammatory conditions in the TME. Consequently, tumors can revert to an immune‐evasive state and regain their growth potential. Thus, there is a need for alternative strategies that can initiate and sustain ICD‐like inflammatory signaling independently of conventional cytotoxic therapies.

Inflammatory conditions that develop during ICD are due to the in situ release of damage‐associated molecular patterns (DAMPs), which are immune‐stimulatory molecules that reside intracellularly and are secreted during cell death [9]. The number of confirmed DAMPs has increased in recent years, ranging from small molecules to proteins, glycoproteins, and proteoglycans [10, 11]. A prominent DAMP is calreticulin [12], a highly abundant endoplasmic reticulum (ER) luminal chaperone protein that is confined to the ER by the ER retention C‐terminal sequence KDEL. During ICD, due to ER stress, a small fraction of calreticulin is secreted and/or enriched at the cell surface. This fraction, termed “ectoCalreticulin,” is recognized by NK cells [13] and macrophages [14], promoting phagocytosis and immune activation. Importantly, calreticulin is only one of many ER‐resident KDEL‐containing proteins with potential immunomodulatory functions. It is therefore expected that the simultaneous release of multiple KDEL proteins, including calreticulin, will further enhance immune stimulation.

The intracellular retention of KDEL proteins is mediated by a conserved quality control system involving KDEL receptors (KDELR1‐3), which facilitate retrograde transport of escaped ER proteins from the Golgi back to ER. KDELRs utilize a sophisticated catch‐and‐release mechanism that exploits the differences in pH between the Golgi and ER to bind the KDEL protein in the Golgi, traffic to the ER, and release the cargo, allowing empty KDELRs to return to the Golgi to resume their gatekeeper function [15]. Although different functions have been attributed to each of the KDELR isoforms, most likely related to their different abilities to signal as GPCR receptors, all three isoforms participate in preventing the secretion of KDEL proteins [15]. This retention system is sensitive to cellular stress. Perturbations such as ER stress or hypoxia can overwhelm or disrupt KDELR‐mediated retrieval, resulting in secretion or surface exposure of KDEL proteins [16, 17, 18, 19, 20]. Notably, studies have suggested that retention of KDEL proteins operates near saturation under basal conditions. As such, a partial disruption by reducing the expression of a single KDELR isoform is sufficient to promote leakage of KDEL‐bearing proteins [16]. Since the mechanism of intracellular retention of KDEL proteins is common to all KDEL proteins, multiple KDEL proteins, not only calreticulin, are displaced *en bloc* from the ER during ICD. This has been shown for protein disulfide isomerases (PDIs), BiP, and gp96 [17, 18, 19, 20].

The role of KDELRs as tumor suppressors has been supported by bioinformatic studies that show a correlation between high expression of KDELR and poor prognosis in various tumor types [21] and a reciprocal correlation with the presence of immune cell infiltration [22]. Based on these findings, we hypothesize that a targeted disruption of KDELR‐mediated retention could mediate the release of a variety of ER‐resident immunomodulatory proteins and thereby mimic or amplify ICD‐like signaling. The current research provides evidence that inhibition of KDELR2 in a small fraction of tumor cells is sufficient to substantially remodel the TME by facilitating a massive infiltration of innate immune cells into the TME. Under these conditions, tumor regression and systemic protection are generated in the absence of conventional cytotoxic interventions. We propose that this approach represents a general strategy to productively initiate the CIC, thereby enhancing the efficacy of immunotherapies.

## 2 Results

### 2.1 Suppression of KDELRs Is Sufficient to Confer Secretion of KDEL Proteins

**FIGURE 1:**
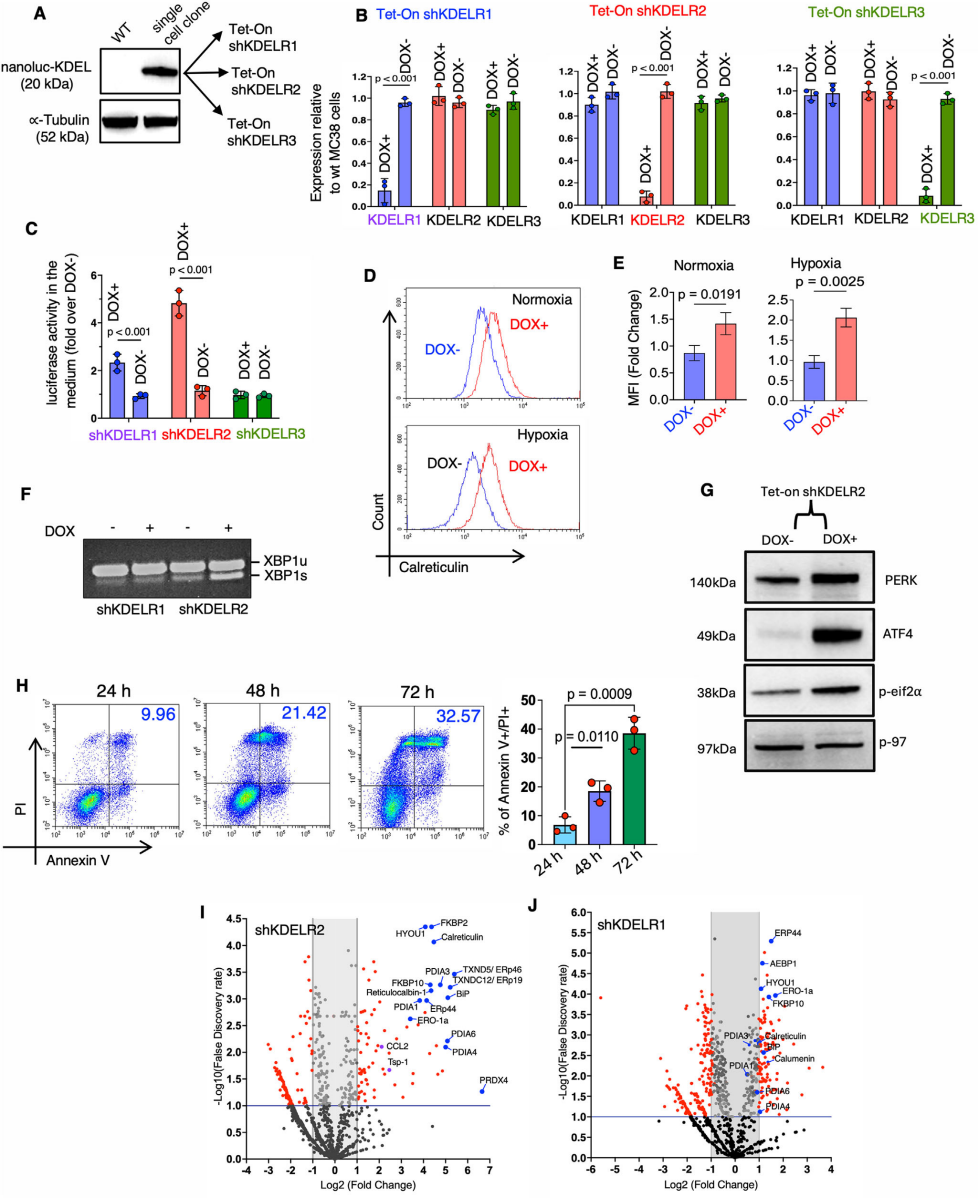
Silencing of KDELR2 is sufficient to induce secretion of secretory KDEL and non‐KDEL proteins. (A) MC38 cells were stably transfected with nanoluc‐KDEL. Cells were then infected with lentiviruses that encode Tet‐On shRNA for KDELR1, KDELR2, or KDELR3. (B) qPCR analysis 24 h after the addition of DOX (10 µM) confirmed activity and specificity of the shRNA sequences. Shown is the mean ± SD of three technical repetitions. (C) Analysis of supernatant luciferase activity following 24 h incubation with DOX. Activity was normalized to activity without DOX. Shown is the mean ± SD of three technical repetitions. (D) Flow cytometry for ectoCalreticulin following 24 h of incubation with DOX under normoxic and hypoxic conditions, quantified (Mean ± SD, *N* = 3) in (E). (F) RT‐PCR analysis of XBP1 mRNA splicing after 24 h of DOX treatment of Tet‐On shKDELR1 and shKDELR2 cells. (G) Immunoblot analysis of the PERK pathway of Tet‐On shKDELR2cells treated with DOX for 24 h. Shown is a representative immunoblot of three independent repetitions. Total eIF2α was analyzed separately from an SDS‐PAGE gel run in parallel. (H) Flow cytometry analysis for late apoptosis of Tet‐On shKDELR2 cells at the indicated times after the addition of DOX. Shown are the means ± SD (*N* = 3). (I and J) Volcano plots for enrichment of proteins in the supernatants of Tet‐On shKDELR2 or Tet‐On shKDELR1 after a treatment for 24 h with DOX, respectively. Data are presented as the mean of triplicates. Statistical significance was determined using Welch's *t*‐test; p < 0.05 was considered significant.

To study the effect of KDELRs on TME, we used the antigenic colon cancer cell line MC38, which was isolated from female C57BL/6 mice [23]. Subcutaneously implanted MC38 cells grow rapidly in naïve C57BL/6 mice. However, growth is inhibited by vaccination [24] and further inhibited by the administration of immune checkpoint inhibitors [25]. Because MC38 is responsive to photodynamic therapy [26], we reasoned that MC38 growth in C57BL/6 hosts would be a suitable model to study the role of KDELR‐KDEL quality control in cancer immunotherapy. To assess which of the three KDELRs is the most dominant in the retention of KDEL proteins, we stably expressed nanoLuc luciferase in MC38 cells, equipped with a signal peptide at the N‐terminus and a KDEL sequence at the C‐terminus. We then introduced a Tet‐On shRNA sequence for each KDELR by lentiviral infection (Figure 1A). Since KDELRs are highly homologous to each other in the amino acid sequence, commercial KDELR antibodies do not distinguish between the different isoforms. Therefore, we used qPCR analyses to confirm shRNA activity 24 h after the addition of doxycycline (DOX) to the medium. As expected, each shRNA was specific for the respective KDELR isoform (Figure 1B). Luciferase activity was measured in the supernatants. DOX‐induced silencing of KDELR2 resulted in the highest increase in luciferase activity in the medium. Silencing of KDELR1 affected less the luciferase activity in the medium than suppression of KDELR2, while the silencing of KDELR3 did not result in any significant effect (Figure 1C). These results were congruent with those observed in SH‐SY5Y cells transfected with siRNA [16]. Calreticulin and other ER proteins adhere to the cell surface upon secretion. Flow cytometry analysis of Tet‐On shKDELR2 cells for ectoCalreticulin showed an increase following the addition of DOX (Figure 1D,E). Because stress conditions promote the accumulation of ectoCalreticulin, ectoBiP, and surface PDIs [27], we examined whether KDELR2 suppression induces ER stress. A hallmark of the UPR is the splicing of XBP‐1 mRNA by the UPR transducer IRE1 [28]. RT‐PCR for XBP1 mRNA demonstrated enhanced splicing after DOX addition for 24 h in Tet‐On shKDELR2 cells (Figure 1F). To assess the activation of additional UPR pathways, we immunoblotted lysates of the Tet‐On shKDELR2 before and after treatment with DOX for 24 h. We observed an increase in amount and size of PERK together with an increase in ATF4 and phosphorylated eIF2α levels, indicative of PERK activation (Figure 1G). We have not analyzed the ATF6 pathway; however, a knockdown of KDELR2 was shown to be correlated with accumulation of ATF6 in the nucleus [29]. We conclude that the silencing of KDELR2 activates a full‐fledged UPR. Since unmitigated conditions of ER stress confer apoptosis [30], we analyzed the level of apoptosis by flow cytometry using Annexin V and propidium iodide (PI) over time. Double positive cells were detected after 48 h of DOX treatment and increased with treatment duration (Figure 1H). To examine if suppression of KDELR2 affects the expression of the other isoforms, we monitored by qPCR the mRNA levels of KDELR1 over time. KDELR2 silencing was effectively maintained following DOX induction, while KDELR1 expression remained unchanged at both 24 and 48 h after addition of DOX, suggesting the absence of compensatory regulation between KDEL receptor family members (Figure S1A). Consistent with sustained KDELR2 inhibition, the nanoLuc KDEL‐reporter activity was significantly elevated at 24, 48, and 72 h after addition of DOX, confirming a persistent disruption of the KDEL ER retrieval system (Figure S1B). Growth retardation of the cells was observed over time (Figure S1C,D), suggesting that the constant suppression of KDELR2 confers irreparable ER stress. Notably, the cytostatic/cytotoxic effect was slower than the release of the KDEL reporter and developed over a few days.

To analyze the effect of KDELR suppression on the cellular secretome, we collected the supernatants for mass spectrometry‐based proteomics. To avoid confounding factors related to serum proteins, cells were cultured for 24 h in OptiMEM medium without serum, supplemented with insulin/transferrin/selenium with and without DOX. At this time point, the viability was minimally affected. Since the suppression of KDELR3 did not affect the secretion of nanoLuc‐KDEL, we only analyzed MC38 cells transduced with shRNA for either KDELR1 or KDELR2. In accordance with the luciferase activity measurements, silencing of KDELR2 resulted in a 20–30 fold increase in the level of KDEL proteins, labeled in blue, such as BiP, PDIA6, PDIA4, and PDIA3 (Figure 1I). KDELR1 suppression was less pronounced (Figure 1J). Notably, the secretome does not include surface‐attached proteins, thus underestimating the total leakage of secretory proteins. Non‐KDEL proteins were also enriched in the supernatants, some with prominent immunomodulatory functions, such as thrombospondin‐1 (TSP‐1) [31] and chemokine C‐C motif ligand 2 (CCL2) [32]. The high mobility group box 1 (HMGB1) is a nuclear DAMP. It is actively secreted or passively released from tumor cells under stress conditions [33]. To examine if KDELR2 inhibition leads to accumulation of extracellular HMGB1, we measured its level in the supernatants of the Tet‐On shKDELR2 cells before and after DOX treatment for 24 h. In two of three experiments, a significant increase in HMGB1 levels was noted (Figure S1E). We conclude that KDELR2 inhibition modestly enriches the extracellular levels of HMGB1, probably due to increased cell death.

**FIGURE 2:**
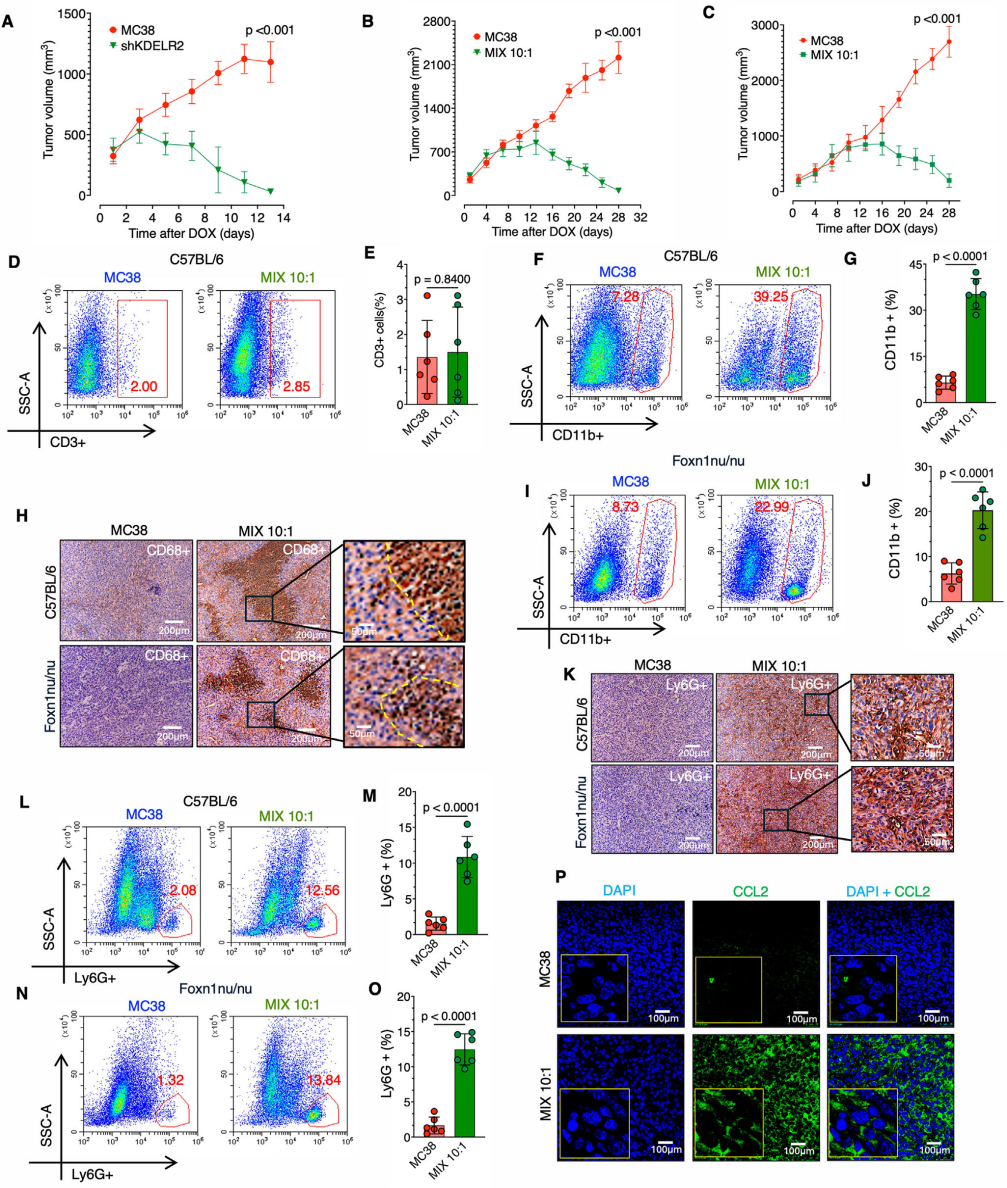
Silencing of KDELR2 in a small percentage of tumor cells confers regression in a T cell‐independent manner associated with the infiltration of macrophages and neutrophils into the TME. (A) Cohorts of 6 C57BL/6 animals were implanted s.c. with MC38 cells on the left and MC38 with Tet‐On shKDELR2 on the right. (A) Mice were put on regular chow and moved to a DOX‐supplemented diet when tumors exceeded 300 mm^3^. Shown are the tumor growth curves, as quantified by a caliper (*N* = 6, mean ± SD). (B) Average growth curves of the tumors, as quantified by a caliper (*N* = 6, mean ± SD). (C) Average growth curves of the tumors, as quantified by a caliper (*N* = 6, mean ± SD). (D) Shown are representative flow cytometry analyses for intratumor T cells. (E) Quantification from 6 different tumors (Mean ± SD). (F) Representative flow cytometry analyses for CD11b+ cells (primarily macrophages) in WT and MIX 10:1 tumors grown in C57BL/6, quantified in (G, *N* = 6, mean ± SD). (H) Immunohistochemistry analysis for CD68‐positive macrophages, demonstrating clusters, in the MIX 10:1 tumors 10 days after placing the C57BL/6 or nude mice on a DOX diet. (I) Same as (H) instead of cells, were implanted into nude mice. (J) Quantification of infiltrated CD11b+ cells into WT and MIX 10:1 tumors (*N* = 6, mean ± SD). (K) Immunohistochemistry analysis for Ly6g+ neutrophils in the MIX 10:1 tumors 10 days after placing the mice on a DOX diet. (L) Representative flow cytometry analyses for neutrophils in WT and MIX 10:1 tumors grown in C57BL/6. Quantified for *N* = 6, mean ± SD (M). (N) Same as (N) instead of hosts were nude mice. Quantified for *N* = 6, mean ± SD (O). (P) Immunohistochemistry analysis for CCL2 in WT and MIX 10:1 tumors 10 days after placing C57BL/6 mice on a DOX diet. Boxed regions (yellow) indicate areas shown at higher magnification in the inset (50µm). Statistical significance was determined using unpaired *t*‐test; p < 0.05 was considered significant.

Suppression of KDELR2 expression in a small fraction of tumor cells results in infiltration of innate immune cells and T cell‐independent tumor regression. To address the role of KDELR2 in tumorigenicity, we subcutaneously challenged C57BL/6 mice in the left flank with wild‐type (WT) MC38 cells and in the right flank with Tet‐On shKDELR2 MC38 cells. Twenty‐four hours before the challenge, the mice were fed either a normal chow diet (DOX‐) or a doxycycline‐supplemented diet (DOX+). Tumor size was assessed throughout the duration of the experiment using calipers. While WT MC38 cells established tumors in both DOX‐ and DOX+ ‐fed mice (Figure S2A,E), Tet‐On shKDELR2 cells established tumors when normal chow was used at similar growth kinetics to WT (Figure S2D), while not establishing tumors when the mice were fed with DOX prior to challenge (Figure S2E). We then tested the effect of KDELR2 suppression once tumors are established. Mice were challenged in both flanks with wt MC38 and Tet‐On shKDELR2 cells. When either of the tumors reached approximately 300 mm^3^, the diet was switched to DOX+. WT MC38 tumors continued to grow. However, Tet‐On shKDELR2 tumors regressed and completely disappeared within two weeks of the diet switch (Figure S2A), consistent with the impeded growth of Tet‐On cells in vitro when exposed to DOX. We then tested whether the suppression of KDELR2 in a small fraction of tumor cells could affect the tumorigenicity of the majority of WT MC38 cells. To this end, we mixed MC38 with Tet‐On shKDELR2 cells prior to inoculation at a 10:1 ratio (denoted as “MIX 10:1”). C57BL/6 mice were challenged with MC38 (left flank) and MIX 10:1 (right flank). When tumors reached approximately 200 mm^3^, the mice were fed a DOX diet. We observed that MIX 10:1 tumors continued to grow for a few days and then regressed. After 28 days, MIX 10:1 was hardly detected, while MC38 tumors continued to grow (Figure S2B). We assumed that the regression was dependent on T cells, since MC38 is antigenic and susceptible to T cell‐mediated responses [34]. Therefore, we repeated the experiment in nude mice lacking T‐cells. Similar to C57BL/6 mice, under the DOX‐supplemented diet, MIX 10:1 tumors underwent regression (Figure 2C, S2C), indicating that the regression does not require T cells.

To address the potential mechanisms of tumor regression, we used histological analysis and flow cytometry to examine MIX 10:1 and wt tumors a week after the mice were placed on a DOX diet. Flow cytometry for T cells indicated a small number of infiltrated cells and no significant difference between MC38 and the MIX 10:1 tumors (Figure 2D,E). A similar phenotype was observed for NK cells (Figure S3). However, a massive amount of CD11b‐positive cells (most likely macrophages) was detected in the MIX 10:1 tumors in some tumor specimens, reaching almost 40% of the total tumor cells (Figure 2F,G). A similar number of infiltrated macrophages was observed in MIX 10:1 tumors isolated from nude mice (Figure 2I,J). Immunohistochemical analyses of the macrophage marker CD68 indicated clusters of macrophages within the tumor tissue in both C57BL/6 and nude mice (Figure 2H). Neutrophils promote an anti‐cancer response by direct killing and by promoting adaptive T‐cell responses [35]. Using Ly6G as a marker, we assessed the presence of neutrophils in the TME. Similar to macrophages, neutrophils were enriched in the MIX 10:1 tumor tissues in both C57BL/6 (Figure 2L) and nude mice (Figure 2N,O). Immunohistochemistry revealed that neutrophils were homogenously dispersed in the tumor tissue (Figure 2K). These data suggest robust infiltration of innate immune cells into the TME, primarily macrophages and neutrophils. This infiltration coincided with regression of tumors a few days after suppression of KDELR2 in as little as 10% of a small subset of tumor cells. CCL2 is a potent chemoattractant for multiple immune cells, including monocytes, macrophages [36, 37] and neutrophils [38]. Since CCL2 levels were enriched in the secretome of Tet‐On shKDELR2, we checked whether this chemokine accumulated in the TME of MIX 10:1 tumors. Immunofluorescence analysis for CCL2 indicated an increase in the TME of MIX10:1 tumors (Figure 2P), suggesting that this chemokine participates in inflammatory conditions that eventually lead to the regression of MIX 10:1 MC38 tumors when KDELR2 is targeted.

**FIGURE 3:**
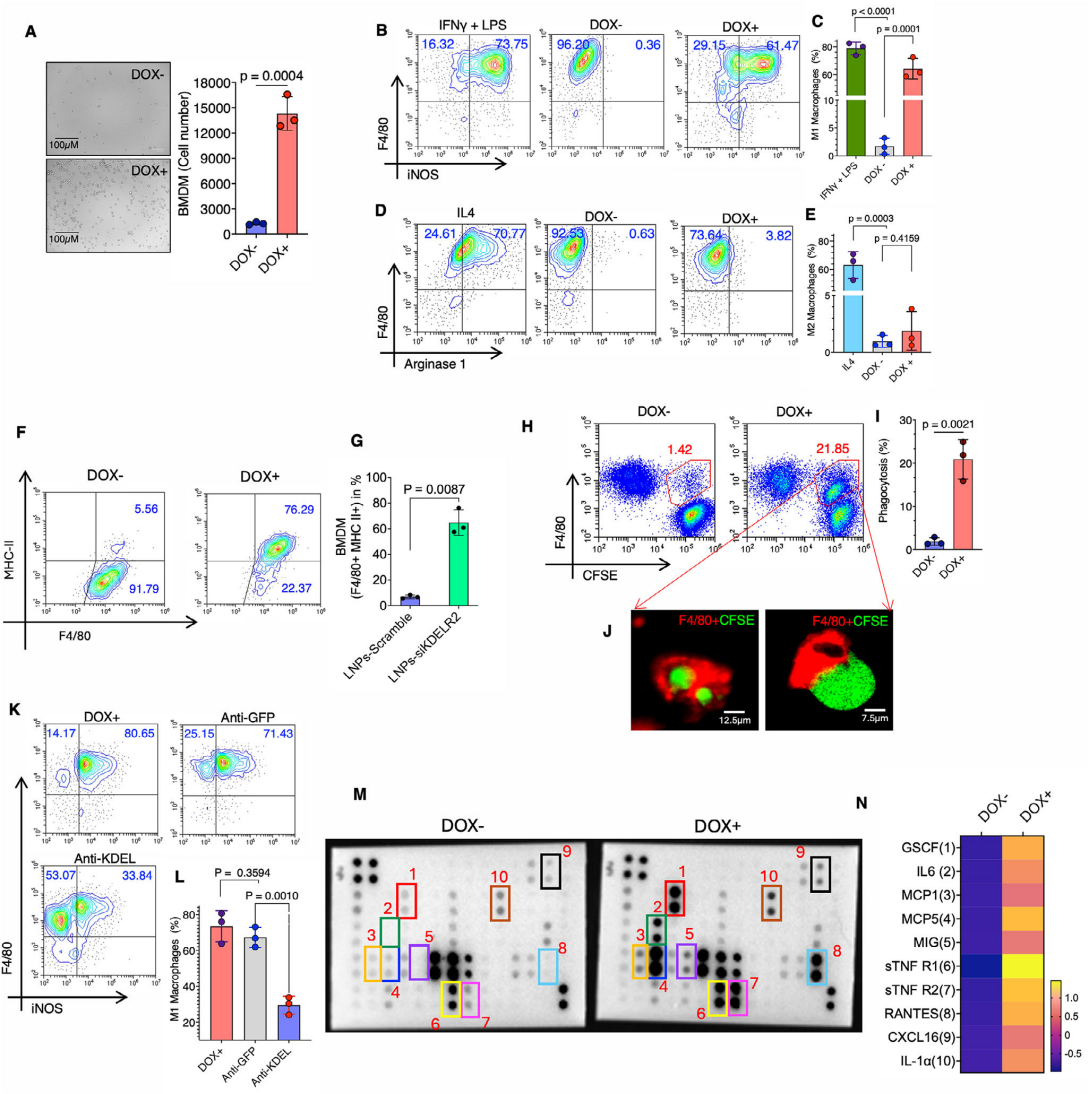
Suppression of KDELR2 promotes macrophage chemotaxis, polarization to M1 and opsonization of tumor cells.(A) BMDM were generated by M‐CSF, polarized to M1 by LPS+IFNγ and incubated with conditioned media of Tet‐On shKDELR2 with and without DOX in a Boyden chamber. Chemotaxis was calculated by counting the cells attached to the separating filter. Shown is a representative image of the filter and quantification (*N* = 3, mean ± SD). (B–E) Naïve BMDM were generated and incubated for 24 h with supernatants of MC38 or Tet‐On KDELR2 cells that were pre‐incubated with DOX. Cells were stained for F4/80, permeabilized and stained for intracellular iNOS (M1), shown in (B)(qunatified for three independent experiments in C, mean ± SD), or arginase 1 (M2), shown in (D, qunatified for three independent experiments in E, mean ± SD). IFNγ or IL4 were used for positive controls of M1 and M2, respectively.(F,H–I) BMDM were polarized to M1, labeled with Alexa647‐anti‐F4/80 and mixed in a 1:5 ratio with CFSE labeled Tet‐On shKDELR2 MC38 cells treated, or not with DOX for 24 h, and quantification from three biological repetitions.(J) Double positive (F4/80+CFSE) cells from the DOX+ group were sorted, cyto‐spin and imaged by epifluorescence.(I) Percent of double positive cells, representing phagocytosis, is shown for three independent experiments, mean ± SD, *N* = 3. (K–L) Naïve BMDM were incubated with supernatants of Tet‐On shKDELR2 preincubated with anti‐KDEL or anti‐GFP. Shown is a representative flow cytometry for M1 polarization and quantification of three repetitions, mean ± SD.(M) Representative cytokine array membranes obtained from supernatants of bone marrow–derived macrophages (BMDMs) treated for 24 h with conditioned media from tumor cells treated with DOX for KDELR2 suppression. Selected cytokines are highlighted on the membrane.(N) Heatmap representation of cytokine expression normalized to positive control spots and transformed to row‐wise Z‐scores, illustrating coordinated upregulation of inflammatory mediators in macrophages following exposure to KDELR2‐conditioned media, which represents KDELR2‐suppressed tumor cells conditioned media reprograms macrophages (BMDM) to a pro‐inflammatory cytokine state. Statistical significance was determined using Welch's *t*‐test; p < 0.05 was considered significant.

Suppression of KDELR2 promotes phagocytosis of tumor cells, macrophage chemotaxis, polarization to M1 in vitro, and release of pro‐inflammatory cytokines and chemokines. At least three subtypes of tumor‐associated macrophages exist [39], of which M1 macrophages directly and indirectly promote anticancer immune responses [40, 41]. To assess whether the suppression of KDELR2 in tumor cells is sufficient to affect macrophage function, we generated bone marrow‐derived macrophages (BMDM), polarized them to M1 with IFNγ + LPS, and analyzed their chemotaxis to supernatants of MC38 Tet‐On shKDELR2 cells treated or not treated with DOX for 48 h. Using Boyden chambers, we found a strong chemotaxis of M1 BMDM in conditioned media collected from DOX‐treated cells (Figure 3A). We then tested whether the supernatants of shKDELR2‐suppressed cells affect macrophage polarization. To this end, naïve BMDM were incubated for 24 h with conditioned media of Tet‐On shKDELR2 pretreated with DOX. Based on the M1 and M2 markers iNOS and Arginase 1, supernatants collected from DOX‐treated Tet‐On shKDELR2 cells conferred an M1 phenotype comparable to the addition of IFNγ + LPS (Figure 3B,C). No M2 macrophages were observed (Figure 3D,E). We next examined whether macrophages are activated upon exposure to the KDELR2‐suppressed tumor secretome. BMDMs treated with conditioned media from KDELR2 suppressed MC38 cells showed a significant increase in expression of class II MHC compared to controls (Figure 3F,G), indicating activation and suggesting acquisition of antigen‐presentation phenotypes. We next investigated whether the suppression of KDELR2 promotes the opsonization of tumor cells by M1 macrophages. We assayed this possibility by flow cytometry in which M1‐polarized BMDM were labeled with Alexa647‐anti‐F4/80 (red) and mixed at a 1:5 ratio with CFSE‐labeled Tet‐On shKDELR2 MC38 cells (green) treated with DOX for 24 h or not. Only in the presence of DOX was a double‐positive population detected, indicative of macrophage/tumor physical interactions (Figure 3H,I). Microscopic analyses of the double‐labeled population showed the presence of tumor cells either adhering to macrophages or actively undergoing phagocytosis (Figure 3J). The secretome of Tet‐On shKDELR2 MC38 cells was enriched in both KDEL and non‐KDEL proteins. To determine whether the polarizing activity of the supernatants required KDEL proteins, we used an anti‐KDEL antibody to deplete the supernatant of KDEL proteins. The anti‐GFP antibody was used as an irrelevant negative control. Using three separate BMDM batches, the removal of KDEL proteins reduced polarization efficiency by more than 50% (Figure 3K,L). We conclude that KDELR2 inhibition enables the polarization of macrophages into M1, promotes their chemoattraction, and opsonizes tumor cells for physical interaction with M1 macrophages. This was brought about by cooperation between KDEL and non‐KDEL proteins. To examine the effect of KDELR2 suppression on inflammatory signals more broadly, supernatants of the BMDM cultures with the conditioned media of the Tet‐On shKDELR2 cells were assayed in a 62‐cytokine/chemokine array. Compared to DOX controls, macrophages exposed to conditioned media from KDELR2‐suppressed cells showed increased levels of several inflammatory mediators, including IL‐6, MCP1/CCL2, MCP5/CCL12, RANTES/CCL5, sTNFR1/R2, CXCL16, and IL‐1α (Figure 3M). Heatmap visualization demonstrated a coordinated enrichment of a broader inflammatory macrophage program in response to the KDELR2‐deficient tumor secretome (Figure 3N). These analyses indicate that exposure of naïve macrophages to the secretome of MC38 cells after KDELR2 inhibition generates a proinflammatory response that results in polarization to M1, chemotaxis, and opsonization of the tumor cells, most likely leading to a productive presentation of tumor antigens on MHC molecules.

### 2.2 Suppression of KDELR2 by a Lipid Nanoparticle Delivery of siRNA Causes Tumor Regression

**FIGURE 4:**
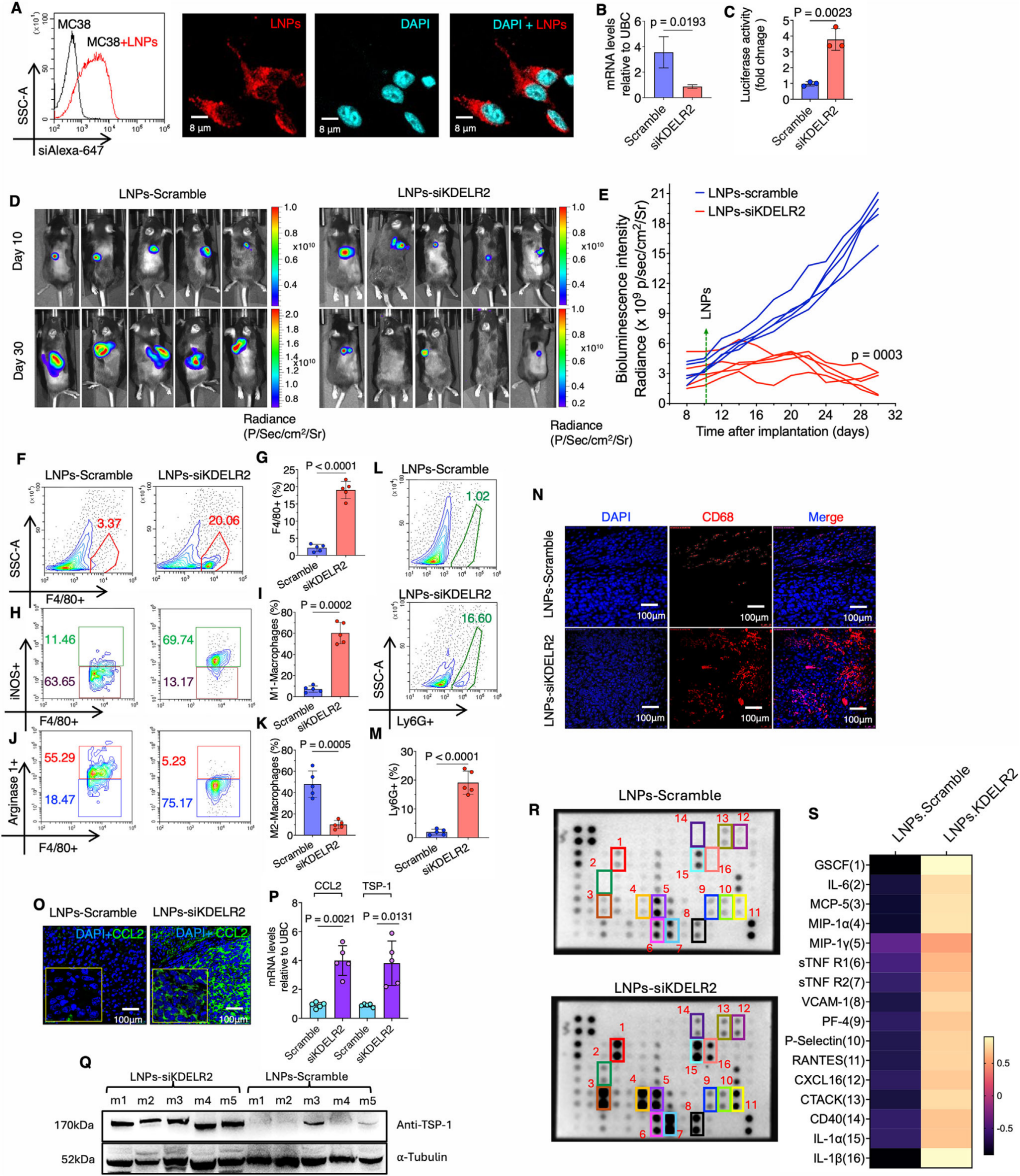
Suppression of KDELR2 by a lipid nanoparticle delivery of siRNA causes regression of MC38 tumors. (A) MC38 cells were incubated with LNPs loaded with siRNA to KDELR2, labeled with Alexa647. Shown by flow cytometry and by epifluorescence microscopy, most MC38 cells have taken up the nanoparticles. (B and C) siKDELR2 LNPs suppress mRNA expression and promote luciferase activity in the supernatants of MC38 /Nanoluc‐KDEL cells. Shown is a representative experiment performed in three technical replicates (*N* = 3, mean ± SD). (D and E) C57BL/6 were inoculated with MC38‐ffLuc cells. When tumors became palpable, LNPs carrying siRNA to KDELR2 or a scramble sequence control were injected into the tumor and growth was followed for an additional three weeks by total body bioluminiscence (*N* = 5). Shown is the bioluminescence signal of every individual mouse (D) and the growth curves of each (E). Tumors were extracted and analyzed by flow cytometry for total macrophages (F quantified in G, *N* = 5, mean ± SD), M1   quantified in I, *N* = 5, mean ± SD), M2 (J quantified in K, *N* = 5, mean ± SD), and neutrophils (L quantified in M, *N* = 5, mean ± SD). (N) Typical immunfluorescence imaging for macrophages. (O) Typical immunfluorescence imaging for CCL2. Boxed regions (yellow) indicate areas shown at higher magnification in the inset (50µm). (P) Total RNA was extracted from each of the five tumors that had been treated with siKDELR2 or a scrambled control LNPs. qPCR analysis shows an elevated level of CCL2 and TSP‐1 mRNA (*N* = 5, mean ± SD). (Q) Western blot analysis of the tumor lysates revealed the upregulation of Tsp‐1 in KDELR2‐compromised mice. (R) Representative cytokine array membranes from tumor lysates of mice treated with LNP‐siKDELR2 or scrambled control, showing differential expression of multiple cytokines. Selected cytokines are highlighted. (S) Heatmap representation of cytokine expression normalized to positive control spots and transformed to row‐wise *Z*‐scores, demonstrating coordinated upregulation of inflammatory mediators in siKDELR2‐treated tumors compared to controls, which represents KDELR2 silencing induces a broad inflammatory cytokine network in the tumor microenvironment in vivo. Statistical significance was determined using Welch's unpaired *t*‐test; p < 0.05 was considered significant.

Because the suppression of KDELR2 in as little as 10% of tumor cells was sufficient to remodel the TME in a profound way, we reasoned that this approach could be recapitulated by conventional nano‐delivery systems of siRNA, which are estimated to deliver their payloads at a similar efficiency [42, 43]. To this end, we constructed siRNA‐loaded LNPs 100–300 nm in diameter with a zeta potential of 50 mV (Figure S4A,B). Encapsulation of the RNA molecules was verified by gel electrophoresis (Figure S4C). These positively charged particles were too large to extravasate easily from the injection site. Previous analyses have demonstrated knockdown efficiency for a few days in vivo [44, 45]. To assess the uptake by MC38 cells, LNPs were labeled with Alexa647. Within 4 h of incubation with MC38 cells that express nanoLuc‐KDEL, most cells were labeled, and the LNPs were observed intracellularly (Figure 4A), most likely in endocytic compartments. qPCR analysis confirmed the reduction in KDELR2 mRNA levels (Figure 4B) and an increase in luciferase activity in the supernatant (Figure 4C), indicating the functional suppression of KDELR2 by the LNPs. We then examined the effects of the LNPs on tumor growth. C57BL/6 mice were inoculated with MC38, which stably expressed firefly luciferase (MC38‐ffLuc). When tumors became palpable (approximately 500 mm^3^), 70 µL of LNPs with siRNA loaded with KDELR2 or a scrambled sequence was injected directly into the tumor. The amount of particles used was enough to affect a small percentage of the tumor cells. We verified this by imaging the Alexa647 LNPs in the tumor tissue and additional organs of the mouse 4 days after injection. At this time point we observed a few LNPs in the tumor tissue and did not observe the LNPs in the lungs, liver, heart, or adjacent lymph nodes (Figure S4D,E). Despite this minute dose, tumor growth was inhibited by the LNP carrying siKDELR2 but continued to grow in the scrambled siRNA control group (Figure 4D,E). We then repeated the experiment, removing the tumors after 20 days. Flow cytometry analysis of tumor tissues indicated the enrichment of macrophages (Figure 4F,G). Analysis of macrophage polarization indicated predominantly M1 macrophages (Figure 4H,I), whereas macrophages in the scrambled control treatment were mostly M2 macrophages (Figure 4J,K). Neutrophils were also observed at higher percentages in siKDELR2‐treated tumors (Figure 4L,M). Histological analysis of tumor sections showed the presence of macrophage clusters (Figure 4N). CCL2 was abundant in tumors in which KDELR2 expression was suppressed (Figure 4O). qPCR analyses of bulk RNA isolated from tumor tissues indicated an increase in CCL2 and TSP‐1 mRNA levels (Figure 4P), suggesting enhanced *de novo* production 20 days after the initiation of KDELR2 inhibition. Western blot analyses of the tumor lysates showed increased TSP‐1 protein levels in the KDELR2‐inhibited tumors (Figure 4Q). To further assess whether KDELR2 inhibition induces a broader inflammatory remodeling within the TME in vivo, cytokine/chemokine profiling was performed using tumor lysates collected from mice treated with siKDELR2 or scramble control LNPs. This analysis revealed elevated expression of multiple inflammatory mediators in KDELR2‐suppressed tumors, including G‐CSF, IL‐6, CCL family, MIP‐1α, VCAM‐1, CXCL16, IL‐1 family, and CD40 (Figure 4R). Heat map analysis demonstrated a consistent inflammatory cytokine signature, which supports that KDELR2‐suppression promotes inflammatory conditions within the TME (Figure 4S).

Myeloid‐derived suppressor cells (MDSCs) suppress multiple anti‐cancer immune responses [46]. MDSCs express Ly6G and Arginase‐1. To examine if the infiltrated neutrophils also contain MDSCs, we analyzed the expression of CD54 (ICAM‐1), associated with N1‐like anti‐tumor neutrophils, and Arginase‐1. Analyzed side by side with macrophages, which display the expected M1 phenotype (Figure S5C–H), KDELR2 suppression resulted in increased Ly6G^+^ neutrophil infiltration, accompanied by a moderate increase of CD54 expression (though not reaching the *p* < 0.05 statistical significance) and a significant reduction in the expression of Arginase‐1 (Figure S5I–N). These data indicate that neutrophils at the N0/N1 state comprise the vast majority of the infiltrated Ly6G^+^ cells.

Injection of LNPs directly into the tumor mass is feasible when the tumor is accessible and the mass is large enough. Because siRNA delivery by LNPs has been shown to be effective in a breast cancer model following systemic injection [47], we examined the efficacy of the approach following intravenous injection. MC38 tumors were allowed to grow to a palpable size, and the LNPs carrying siRNA for KDELR2 or a scrambled control were injected three times. While the tumor growth was moderately reduced, despite the multiple doses (Figure S6A, B), M1 polarization and recruitment of neutrophils to the TME were observed (Figure S6C–N). Of note, the injected LNPs did not contain a targeting moiety. These findings demonstrated the potential of LNPs carrying siRNA to KDELR2 to remodel the TME, provided that efficient targeting is achieved.

The similar phenotype of KDELR2 inhibition by the Tet‐On system and by LNP delivery of siRNA prompted us to examine whether suppression of KDELR2 in human cancer cells also promotes macrophage activation and polarization. To this end, we treated human breast cancer cells, HCC1806 with LNPs loaded with siKDELR2 or scrambles. Cells were labeled with red fluorescent dye and mixed human macrophages derived from peripheral blood of a healthy donor and polarized into M1. Similar to the mouse system, suppression of KDELR2 opsonized the human cancer cells (Figure S7A,B). We then tested if the conditioned media of the human cells are sufficient to confer polarization of naïve macrophages. Using the M1 marker CD86, more than half of the macrophages were polarized to M1 after incubation with the conditioned media (Figure S7 C and D). Polarization to M2 was not observed (Figure S7E,F). Based on these ex vivo experiments, we conclude that LNP‐mediated delivery of siKDELR2 can also be used to stimulate the recognition of human cancers by the immune system.

### 2.3 Suppression of KDELR2 Causes the Regression of B16F10 Melanoma

**FIGURE 5:**
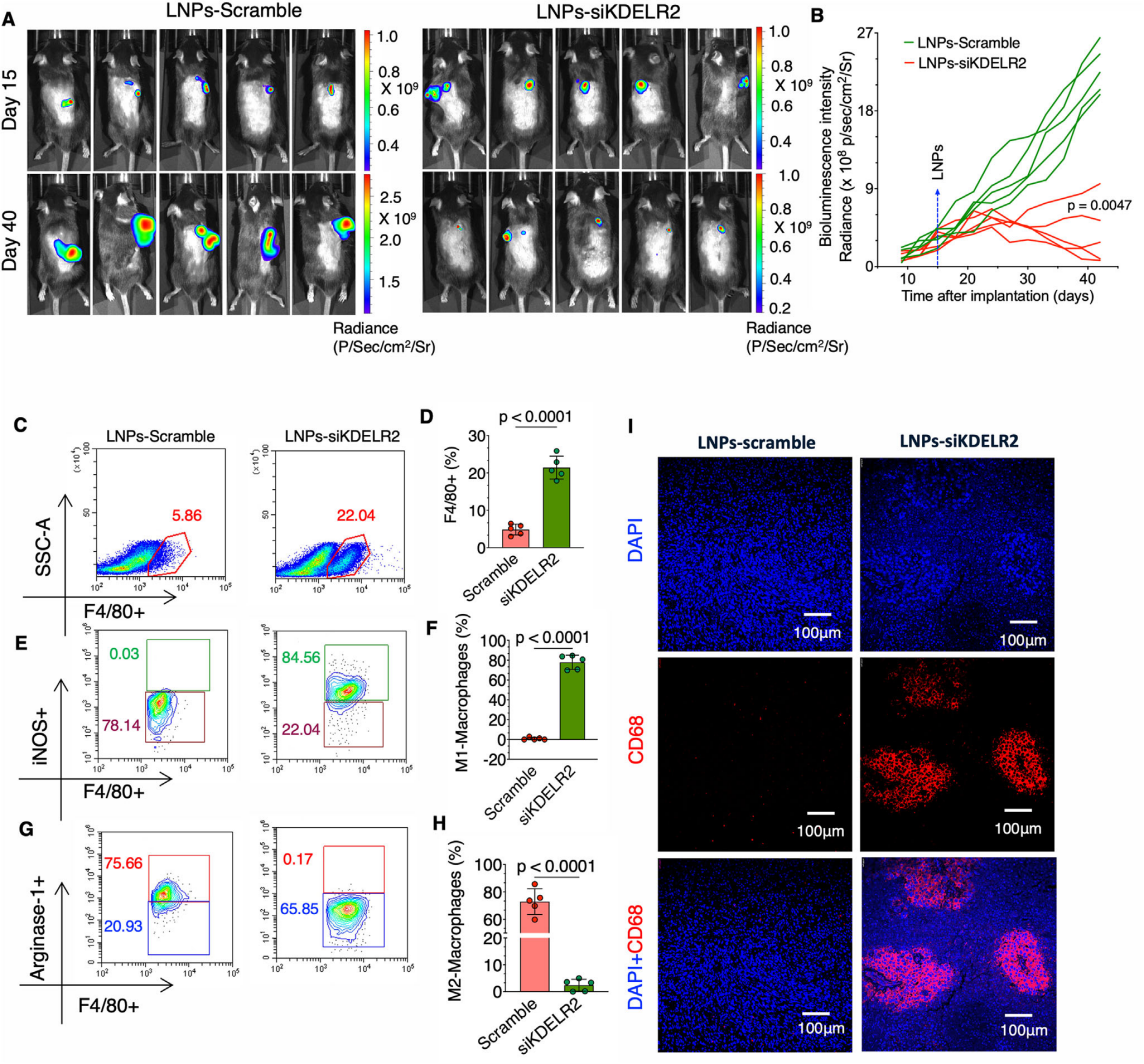
Suppression of KDELR2 by a lipid nanoparticle delivery of siRNA causes regression of B16F10 tumors. C57BL/6 males were inoculated with B16F10‐ffLuc cells. When tumors became palpable, LNPs carrying siRNA to KDELR2 or a scramble sequence control were injected into the tumor, and growth was followed by total body bioluminiscence (*N* = 5). Shown is the bioluminescence signal of every individual mouse at the indicated days after inoculation (A) and growth curves (B). Tumors were extracted and analyzed by flow cytometry for total macrophages (C quantified in D, *N* = 5, mean ± SD), M1 (E quantified in F, *N* = 5, mean ± SD) and M2 (G quantified in H, *N* = 5, mean ± SD). (I) Immunofluorescent images for macrophages of representative tumors from the two cohorts demonstrating the clusters of macrophages following KDELR2 silencing. Statistical significance was determined using Welch's unpaired *t*‐test; p < 0.05 was considered significant.

MC38 is an antigenic tumor that is efficiently curtailed by T cells. B16F10 melanoma cells establish aggressive tumors that are poorly recognized by T cells, owing to their low MHC class I expression [48] and high expression of PD‐L1 [49]. To examine whether KDELR2 inhibition by LNPs affects the growth of non‐immunogenic tumors, C57BL/6 mice were challenged subcutaneously with B16F10 cells that stably expressed firefly luciferase. Fifteen days after implantation, when tumors were readily observed, 70 µL of LNPs carrying siRNA to KDELR2 or a scrambled sequence were injected into the tumor. Tumor growth was monitored using bioluminescence. Injection of KDELR2 siRNA LNPs resulted in retarded growth followed by regression, while the tumors continuously progressed in the control group. This was demonstrated in images of individual mice taken before LNP injection and at day 40 post‐implantation, before euthanasia (Figure 5A). The bioluminescence signal of each mouse over time is shown in Figure 5B. A separate group of mice was used for TME analyses. Tumors were isolated at day 30 post‐implantation, 15 days after the administration of LNPs, and analyzed by flow cytometry for macrophages and histology. Similar to what was observed for MC38, macrophage abundance increased in the TME upon KDELR2 suppression (Figure 5C,D), and almost all exhibited an M1 phenotype (Figure 5E,F). The predominant M2 phenotype was observed in the control group (Figure 5G,H). Histological analysis showed clusters of macrophages in the siKDELR2 group (Figure 5I). We conclude that remodeling the TME in response to KDELR2 suppression is similar for hot and cold tumors.

### 2.4 Suppression of KDELR2 Causes the Regression of Orthotopically Implanted Tumors

**FIGURE 6:**
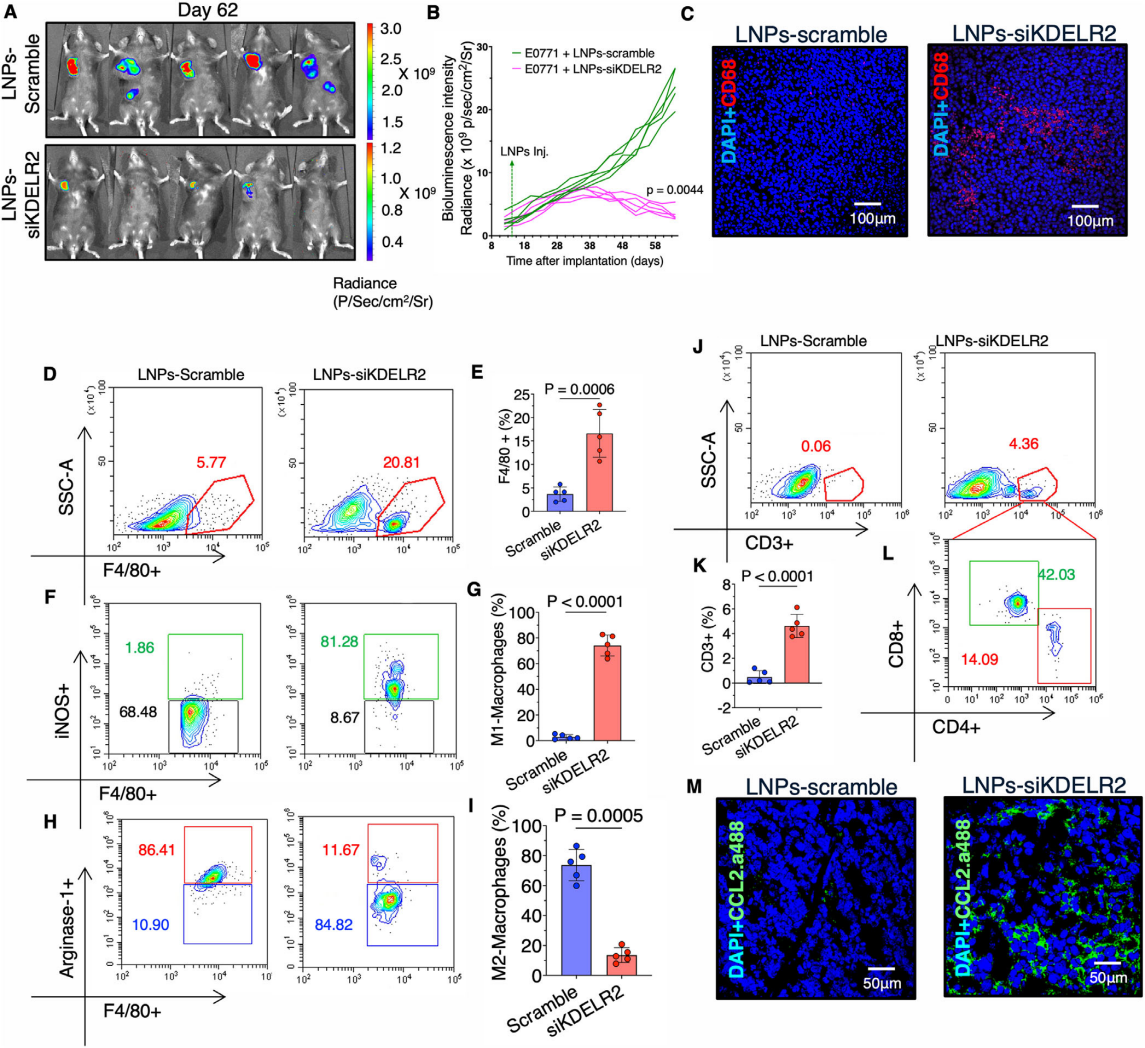
Suppression of KDELR2 by a lipid nanoparticle delivery of siRNA causes regression of orthotopic breast cancer tumors. C57BL/6 females were inoculated into the mammary fat pad with E0771‐ffLuc cells. When tumors became palpable, LNPs carrying siRNA to KDELR2 or a scramble sequence control were injected into the tumor and growth was followed by total body bioluminiscence (*N* = 5). Shown is the bioluminescence signal of every individual mouse at day 62 after inoculation (A) and growth curves (B). (C) Immunofluorescent images of tumors for macrophages (CD68). Flow cytometry analyses of the tumors show an increase in infiltrating macrophages (D quantified in E, *N* = 5, mean ± SD) and polarization into M1 (F quantified in G, *N* = 5, mena ± SD), while the macrophages were polarized into M2 in the scrambled control group (H quantified in I, *N* = 5, mean ± SD). (J) Infiltrated T cells were measured by flow cytometery, qantntified in (K, *N* = 5, mean ± SD). Analysis of CD4 and CD8 indicated that most TILs were CD8‐positive (L). (M) imunofluorescence analysis for CCL2 demonstrates higher levels in the tumors treated with siKDELR2 LNPs than the controls. Statistical significance was determined using Welch's unpaired *t*‐test; p < 0.05 was considered significant.

We next wondered whether tumors growing in tissues other than the skin would also exhibit infiltration of innate immune cells after KDELR2 suppression. To address this, we stably expressed firefly luciferase in an E0771 triple‐negative breast cancer cell line. The cells were implanted into the mammary fat pads of C57BL/6 female mice. When reaching a size of 300–500 mm^3^, 70 µL of LNPs carrying siRNA to KDELR2 or a scrambled sequence was injected into the tumor. While the control tumors continued to grow, the E0771 tumors treated with siRNA against KDELR2 grew slower and then started to regress (Figure 6A). Of note, regression was slower than that for MC38 or B16F10 tumors. Moreover, 62 days after inoculation (47 days after LNP injection), the tumors were still detected (Figure 6B). When analyzed histologically 30 days after administration of LNPs, infiltration of macrophages was observed; however, big clusters were not observed (Figure 6C). Flow cytometry estimated the percentage of macrophages to be approximately 15% (Figure 6D,E). As observed for MC38 and B16F10 tumors, infiltrated macrophages were polarized primarily to M1 after KDELR2 silencing (Figure 6F,G) and to M2 in the control group (Figure 6H,I). In contrast to MC38 tumors, perhaps due to the long time needed for regression, T cells were enriched in primary orthotopically implanted E0771 tumors (Figure 6J,K), and most were CD8‐positive (Figure 6L). CCL2 was enriched in siKDELR2 tumors (Figure 6M). We conclude that inhibition of KDELR2 remodels the TME to inhibit tumor growth in the mammary fat pad, although with slower kinetics.

### 2.5 Regression of KDELR2‐Suppressed Tumors Reduces Tumor Growth Upon Rechallenge, Associated With Enhanced T Cell Infiltration

**FIGURE 7:**
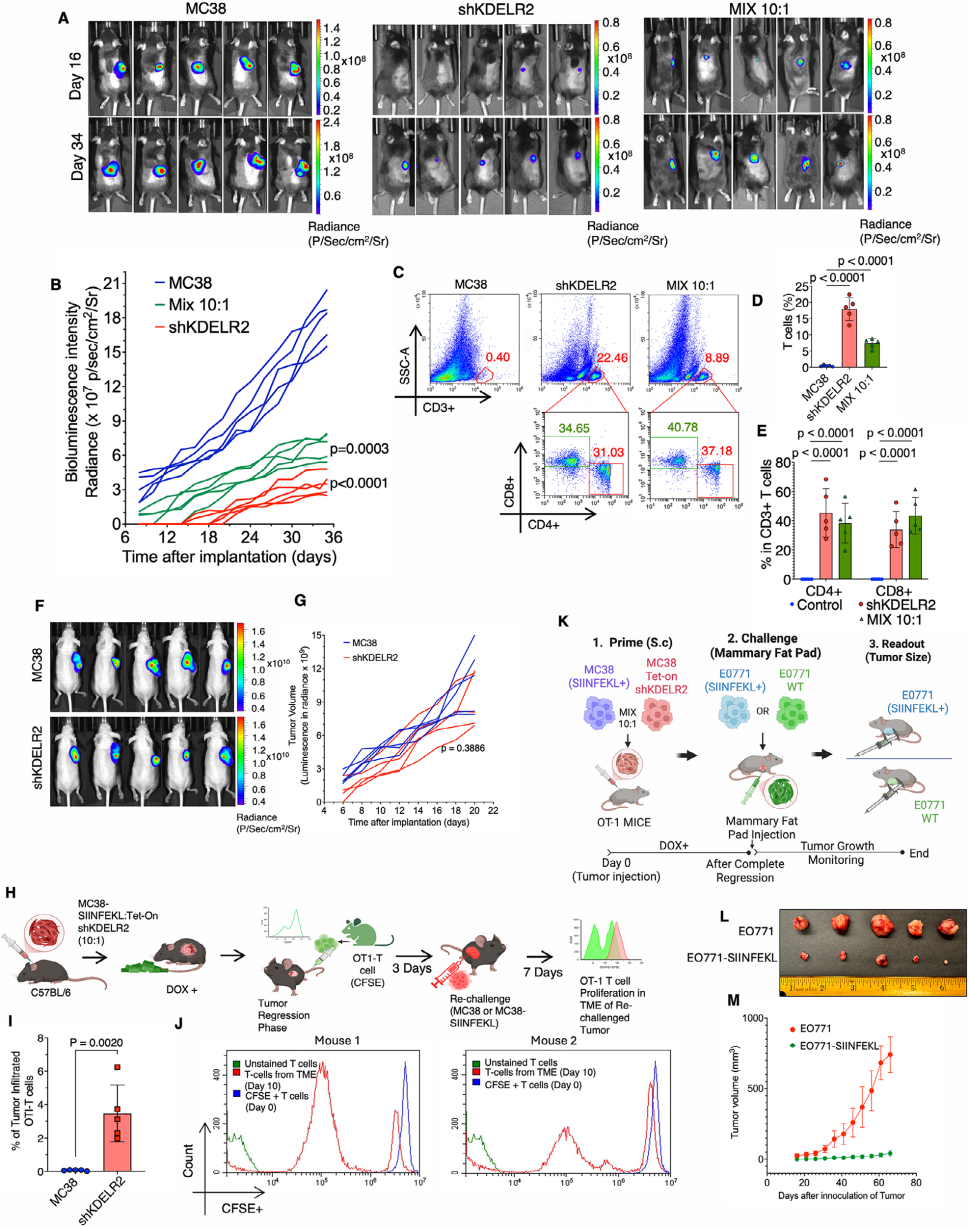
Suppression of KDELR2 induces T cell priming and a protective T cell response. (A) C57BL/6 mice were implanted with MIX 10:1 or Tet‐On KDELR2 cells. Tumors were allowed to progress to 800 mm^3^, then mice were placed on a DOX diet until tumors were no longer observed. Two weeks later, naive mice or mice in which tumors had been regressed were challenged with MC38‐ffLuc cells and followed up to 34 days. Shown are the bioluminiscence images of five mice in each group at the indicated days post inoculation. (B) Blue lines represent the tumor growth of MC38 in naïve mice. In green after MIX 10:1 regression and in red is growth after Tet‐On shKDELR2 regression. (C) At the end of the experiment tumors were isolated and analyzed by flow cytometry for total T cells and CD4 and CD8 distribution. Total T cells are quantified in (D, *N* = 5, mean ± SD) and CD4/CD8 distribution in (E, *N* = 5, mean ± SD). (F and G) Nude mice were treated as in A. Shown is the similar bioluminiscence and similar growth kinetics following tumor regression. (H) A schematic layout of OT‐I transfer. C57BL/6 mice were challenged s.c. with a MIX 10:1 of MC38 that express the SIINFEKL antigen and MC38 Tet‐On shKDELR2. Tumors were allowed to develop and then mice were put on a DOX diet. After a week naive CFSE‐labeled OT‐I T cells were adoptively transferred. Three days later mice were challenged with either wt MC38 or MC38 SIINFEKL. (I) Seven days later, while mice are on a DOX diet, tumors were isolated and analyzed by flow cytometry for OT‐I T cell proliferation. Shown are the levels of infiltrated OT‐I cells in wt and SIINFEKL‐expressing tumors (*N* = 5, mean ± SD). (J) CFSE dilution pattern of OT‐I T cells in two MC38 SIINFEKL tumors. (K) A schematic layout of KDELR2‐driven tumor regression leading to antigen‐specific T cell memory and systemic protection against tumor growth. OT‐I mice were challenged s.c. with a MIX 10:1 of MC38 that express the SIINFEKL antigen and MC38 Tet‐On shKDELR2. Following complete regression, mice were challenged in the mammary fat pad with E0771 or E0771 cells that express the SIINFEKL antigen. Shown is the tumor size after removal at the end of the experiment and (L) the average growth ± SD, as measured with a caliper (*N* = 5). Statistical significance was determined using Welch's unpaired *t*‐test; p < 0.05 was considered significant.

T cells are essential for maintaining immunosurveillance and protection from tumor recurrence [50]. To address whether ICD conditions induce T cell activation, we analyzed the memory response two weeks after regression of the MIX 10:1 tumors. Using the memory markers CD44 and CD62L, we observed an elevation of central and effector T cells in the spleens (Figure S8). To assess functionality, we examined the growth of MC38 cells upon a second challenge after regression of KDELR2‐silenced tumors. C57BL/6 mice were subcutaneously inoculated with MIX 10:1 or Tet‐On shKDELR2 cells. Tumors were allowed to develop to 200 mm^3^, and then the mice were placed on a DOX diet until the tumors were no longer observed. Two weeks later, while still on the DOX diet, a second challenge with MC38 cells was performed. To ensure that the emerging tumors were not derived from dormant primary tumor cells, the second challenge was performed with MC38‐ffLuc, and tumor growth was assessed by whole‐body bioluminescence. Re‐emerging tumors were confined to the injection location, without evidence of metastasis. When compared to the growth of MC38 cells in naive mice (blue lines), a significant decrease in tumorigenicity was observed (Figure 7A,B, compare green to blue lines). Shown for individual mice, a stronger inhibitory effect was seen when only the Tet‐On shKDELR2 was used for the first challenge (Figure 7B, red lines), suggesting that inhibition of KDELR2 has a “dose”‐dependent effect against a re‐challenge. Analysis of the tumors 34 days after rechallenge of C57BL/6 mice indicated the presence of T cells (Figure 7C,D) composed of both CD4 and CD8 subtypes at roughly even proportions (Figure 7C,E), consistent with previous studies showing a crucial role of T cells in controlling MC38 growth [51]. Similar to what had been observed for the primary MC38 MIX 10:1 tumors, the rechallenged MC38 tumors were enriched for macrophages, predominantly M1 macrophages (Figure S9), without any additional immune stimulation. To assess whether the retardation in growth was dependent on T cells, we repeated the experiment in nude mice. In this host, MC38‐ffLuc growth was not significantly affected by regression of Tet‐On shKDELR2 cells (Figure 7F,G). These data suggest that the regression of tumors by the inhibition of KDELR2 initiates a systemic anti‐cancer response that may protect against tumor dissemination. To address the possibility of T cell priming during the regression of primary tumors, we generated MC38 cells that express SIINFEKL, an ovalbumin‐derived immunodominant H‐2K^b^ MHC class I‐restricted epitope. This epitope is recognized by T cells in OT‐I TCR transgenic mice [52]. We purposely expressed the SIINFEKL peptide using the PresentER vector, which deposits the minimal epitope directly in the ER, obviating proteasomal degradation and TAP‐dependent transport [53] and avoiding confounding issues of stress‐related effects on the MHC class I presentation machinery. MC38‐SIINFEKL cells were mixed with Tet‐On shKDELR2 cells at a 10:1 ratio and subcutaneously implanted into C57BL/6 mice. When the tumors were palpable, the mice were fed a DOX diet for a week. Before complete regression of the primary tumor, CFSE‐labeled naïve CD8+ T cells isolated from the spleens of OT‐I mice were adoptively transferred. Three days later, while still on the DOX diet, the mice were rechallenged on the other flank with either wt MC38 or MC38‐SIINFEKL. One week later, the tumors were removed and analyzed by flow cytometry (Figure 7H). While OT‐I T cells were not evident in any of the analyzed wild‐type MC38 tumors, we detected OT‐I T cells at a frequency of approximately 3% of the total MC38‐SIINFEKL tumor cells (Figure 7I). Proliferation was observed with CFSE dilution, as shown for two different tumors (Figure 7J). To examine if the primed T cells protect against tumor growth, we challenged OT‐I mice with MC38‐SIINFEKL cells mixed in a 10:1 ratio with the Tet‐On shKDELR2 cells. Tumors were allowed to regress under the DOX diet and then challenged in the mammary fat pad with E0771 cells, or E0771 that stably express SIINFEKL (Figure 7K). Growth of the E0771‐SIINFEKL was slower than the growth of E0771 WT cells (Figure 7L,M). This indicates a priming of tumor‐recognizing T cells without active vaccination that provides systemic protection.

## 3 Discussion

Except for platelets, in which PDI proteins are stored in secretory granules and secreted upon activation [54], in most cells, KDEL proteins are released in an uncontrolled manner, often due to ER stress [55]. This indicates that, in most cells, the retention of KDEL proteins in the ER is inefficient and subject to leakage. Three potential mechanisms have been ascribed to the secretion of KDEL proteins: (i) secretion due to saturation of KDEL receptors (KDELRs) [16]; (ii) inhibition of the interaction of the KDEL sequence with the cognate receptors [56]; and (iii) trafficking of the KDELRs to the plasma membrane, escorting their cargo for secretion [55]. While these mechanisms probably operate in tandem, our data support a near saturation of the KDEL‐KDELR interactions in MC38 cells. For both KDELR1 and KDELR2, downregulation was sufficient to induce the release of KDEL proteins.

The relative abundance of the KDELR isoforms varies among cells [57]. We decided to silence each KDELR isoform separately to determine the dominant isoform in MC38 cells. Our data show that KDELR2 is the dominant gatekeeper isoform in this cell type, similar to what has been found in various human cells [16, 58]. While not tested here, a standing question is whether the targeting of KDELR isoforms will provide stronger ICD conditions than the silencing of only one isoform. For optimal immunomodulatory bystander effects, a balance between cell viability and secretion should be considered. Most MC38 cells survived in vitro for up to four days after the initiation of KDELR2 knockdown. The silencing of the three KDELRs together might compromise cell viability too rapidly, which would be insufficient to allow the accumulation of DAMPs in the TME. Defining the best combination and timing of KDELR silencing should be performed to optimize TME remodeling.

The secretome of MC38 shKDELR2 cells was enriched in KDEL and non‐KDEL proteins. This is not surprising, as KDEL proteins, primarily those engaged in protein folding, are required for the retention of non‐KDEL proteins. One of the proteins that caught our attention was TSP‐1, which was enriched in the KDELR2 secretome by more than four‐fold. TSP‐1 is retained in the ER in a PDIA3‐dependent manner [59, 60]. Once released, TSP‐1 interacts with macrophages and promotes the secretion of IL‐1β, a strong pro‐inflammatory cytokine [61]. It is therefore feasible that the modulation of the TME by KDELR2 inhibition is a result of the cooperative activity of the KDEL and non‐KDEL proteins. The supernatants of KDELR2‐inhibited MC38 cells conferred macrophage polarization. This attribute has been ascribed to proinflammatory cytokines such as TNF‐α, IL‐1β, IL‐6, and IL‐12 [62]. KDEL proteins have not been documented to directly affect polarization. Therefore, it was unexpected that the depletion of KDEL proteins from the supernatants only would reduce the polarization efficiency. This can be due to the action of KDEL proteins directly on macrophages or, most likely, that KDEL proteins physically interact with cytokines, and upon depletion, a portion of the cytokines is also removed. This signifies that KDEL proteins with chaperone activity, such as BiP, gp94, and calreticulin, when released in situ, may carry proinflammatory cytokines and promote their activity. We envision a positive feedback loop in which a small number of tumor cells release KDEL and non‐KDEL proteins that initiate the infiltration of innate immune cells, macrophages, and neutrophils. Once in the TME, these immune cells are activated, polarized, and invigorate the infiltration by releasing chemokines and cytokines. This model may explain macrophage clusters. We assumed that neutrophils were not observed in the clusters owing to their higher mobility, allowing them to disperse through the tumor tissue. In support of this model, the TME is inundated with CCL2, a seminal chemokine for innate immune cells [63]. Our data demonstrate robust immune remodeling upon KDELR2 inhibition. The underlying mechanisms are likely multifactorial. Although CCL2 is strongly elevated, a broader inflammatory network, including IL‐6, CCL family chemokines, and the IL‐1 family, is likely to be involved. These mediators collectively contribute to the recruitment and activation of innate immune cells, which then recruit and activate T cells. While our data indicate the potential of macrophages to be the APCs, we cannot exclude contributions from additional APCs such as DCs, which were not extensively characterized in this study.

The effect of KDELR2 inhibition in as little as 10% of the tumor cells was sufficient to cause complete regression of MC38 tumors. The growth of a second challenge was inhibited, but not prevented, even for antigenic tumors, such as MC38. The adoptive transfer of OT‐I T cells and the use of OT‐I mice as hosts indicate priming and functional activation. Priming of T cells requires an ordered lymphoid structure. Tertiary lymphoid tissues can be generated inside organs under prolonged chronic inflammatory conditions [64]. Although indirect evidence for T‐cell priming within tumor tissues exists [65, 66], it is unlikely that T‐cell priming occurred in situ. A more likely scenario is the cross‐presentation of tumor antigens by APCs at the local draining lymph nodes. This can be mediated by infiltrated M1 macrophages and/or DCs, suggesting in‐and‐out trafficking. We propose that the enhanced permeability of the tumor tissue provoked by inflammatory conditions reprograms the TME to be non‐permissive for tumor growth. These features seem to operate irrespective of T‐cell antigenicity. We propose that tumors that are unresectable but approachable for direct administration of RNA delivery systems, siRNA or antisense, which mildly respond to immunotherapy, would benefit most from this approach. This includes primary liver cancer or large liver metastases, which can be assessed using catheterization. Another potential application is metastatic large cell neuroendocrine carcinoma of the lung, which is a rare and aggressive type of lung cancer accessible by endoscopy [67], and sarcomas. Supported by the similar response of human macrophages, we propose that the silencing of KDELRs can be leveraged to directly initiate CIC, overriding the need for therapy to induce ICD. To maximize the effect, a combination of KDELR inhibition with immune checkpoint inhibitors or CAR‐T cells should be considered to sustain productive CIC. This study describes a comprehensive characterization of immune remodeling induced by KDELR2 inhibition. Several aspects warrant further investigation using high‐resolution approaches. In addition, while systemic delivery of KDELR2 demonstrated measurable efficacy and recapitulated key immune features, the overall effect did not result in regression. Optimization of delivery strategies should be employed to enhance the efficacy. This includes modifying the LNPs with tumor‐targeting ligands, such as a cyclic Arginine‐Glycine‐Aspartic (RGD) peptide, single‐chain antibodies, and so forth [68].

A further understanding of the temporal signaling axes may lead to the design of stronger and more durable effects that can synergize with the various immunotherapeutic modalities. These directions will help refine the mechanistic framework and further support the translational potential of KDELR targeting.

## 4 Materials and Methods

### 4.1 Sex as a Biological Variable

To avoid confounding immunological responses due to a gender mismatch, we decided to study the female cell lines MC38 and E0771 in female mice and B16F10 melanoma cells in male mice. Experiments were not done blindly.

### 4.2 Mice

Breeding pairs of C57BL/6J and OT‐I (C57BL/6‐Tg(TcraTcrb)1100Mjb/J) mice were purchased from Jackson Laboratories. Colonies were established for experiments. Mice were housed in IVC cages, with specific rules for breeding, handling, and preventing overcrowding. The Institutional Animal Care and Use Committee (IACUC) at CWRU oversees all animal use, ensuring compliance with these standards and regulations. CWRU's animal research follows guidelines from the PHS Policy, the Animal Welfare Act, and AAALAC standards, with specific SOPs in place. All experimental protocols were performed in accordance with the institutional guidelines, approved by the IACUC of CWRU, protocol # 2023‐0052.

### 4.3 Cell Lines

All cell lines were routinely checked for mycoplasma contamination by LookOut Mycoplasma PCR Detection Kit (Millipore Sigma, cat# MP0035). MC38 cells were purchased from Kerafast (Shirley, MA). E0771 cells were purchased from ATCC. HCC1806 cells were obtained from Dr. William Schiemann (CWRU, OH, USA). Cells were not directly authenticated. pcDNA3, which encodes the nanoLuc‐KDEL, was provided by Dr. Mohammad Mahameed (Hebrew University, Israel) and was used to stably express MC38. shRNA sequences targeting KDELRs were cloned into Tet‐pLKO‐puro (Addgene, cat# 21915). Oligonucleotide sequences were:

KDELR1: CCGGTGGTGTTCACTGCCCGATATCCTCGAGGATATCGGGCAGTGAACACCATTTTTG

KDELR2: CCGGGACCATTCTCTACTGCGACTTCTCGAGAAGTCGCAGTAGAGAATGGTCTTTTTG

KDELR3: CCGGGCAAGCTGTAAGCAGTCCAAACTCGAGTTTGGACTGCTTACAGCTTGCTTTTTG

The cells were infected with lentiviruses carrying shRNA sequences. Selection was performed using 1 µg/ml puromycin. Firefly luciferase was expressed in MC38 and E0771 cells by lentiviral infection, using pLX313‐Firefly luciferase. B16F10 melanoma cells expressing firefly luciferase were kindly gifted by Dr. Serge Fuchs (U Penn, Philadelphia, PA, USA). All cells were maintained in DMEM supplemented with 10% fetal bovine serum (Corning, MT35010CV), 2 mM L‐glutamine (Thermo Fisher Scientific, 25030081), 1% penicillin‐streptomycin solution (Thermo Fisher Scientific, 15070063), and 1 mM sodium pyruvate (Thermo Fisher Scientific, 11360070).

### 4.4 PCR and qPCR

Total RNA was extracted using QIAzol Lysis Reagent (Qiagen cat#79306), according to the manufacturer's protocol. 1 µg of it was reverse‐transcribed using the iScript cDNA Synthesis Kit (Bio‐Rad, cat# 1708891) according to the manufacturer's instructions. qPCR was performed using SsoAdvanced Universal SYBR Green Supermix (Bio‐Rad, cat#1725270) on a CFX96 Touch Real‐Time PCR machine (Bio‐Rad). PCR for XBP1 mRNA splicing was performed using the REDExtract PCR ReadyMix (R4775, Sigma‐Aldrich), as previously described [69]. UBC was used as a normalizer.

Real Time Primers:

mKDELR1‐F GATGGCAACCACGACACTTTCC, mKDELR1‐R GGCAAGATAGCCACTGACTCCA

mKDELR2‐F CGATACCTTCCGAGTGGAGTTC, mKDELR2‐R CCAGGTAGATGGAGAAGGTCCA

mKDELR3‐F  GTGACTGGCCTTTCCTTTCT, mKDELR3‐R CACCGGGAGGCTTAATTTCT

mUBC‐F CAGCCGTATATCTTCCCAGACT, mUBC‐R CTCAGAGGGATGCCAGTAATCTA

### 4.5 Secretome Analyses by Mass Spectrometry

The cells were cultured in OptiMem supplemented with insulin/transferrin/selenium for 24 h in the presence or absence of 10 µM doxycycline. Cells were centrifuged, and the supernatants were concentrated with Centricons with a cutoff of 3 kDa (Millipore #UFC500396), dried in SpeedVac, and reconstituted in 25 µL of 6 M urea/Tris buffer. The protein samples were reduced with 120 mM DTT for 15 min at 55°C and then alkylated with 500 mM iodoacetamide in the dark for 20 min at room temperature. The proteins were concentrated by overnight acetone precipitation at −20°C. Protein pellets were air‐dried for 10 min and dissolved in 40 µL 50mM tri‐ethyl ammonium bicarbonate (TEAB) with 1 µg sequencing‐grade trypsin and incubated at 37°C overnight. Digestion was terminated by adding TFA at a final concentration of 1%. The peptide samples were dried in a SpeedVac, reconstituted in 100 µL of 0.1% formic acid, and filtered through a 0.22 µm MilliporeSigma Ultrafree‐MC Centrifugal Filter (#UFC30GVNB) before LC‐MS/MS.

LC‐MS/MS analysis: The samples were analyzed by LC‐MS using a timsTOF Pro2 instrument (Bruker) equipped with a NanoElute UHPLC system. A 5 µL aliquot of each digest was injected onto a ThermoScientific (0.5 × 5 mm) Acclaim PepMap C_18_, 5‐µm, trapping column. Liquid chromatographic elution was performed using a flow rate of 0.3 µl/min on a reverse‐phase column (ReproSil AQ C18, 75 µm x 150 mm, 1.9‐µm 120‐Å). Peptide elution was performed using a binary gradient of mobile phase A (0.1% formic acid) and mobile phase B (0.1% formic acid in acetonitrile). Each sample was analyzed using a linear gradient starting at 2% B at 0 min and increasing to 35% B in 30 min, followed by an increase to 90% B in 2 min, and then holding at 90% for 5 min before re‐equilibration at 2% B. The electrospray voltage was 1500 V. PASEF‐DDA was used for peptide identification. MS1 scans were carried out with a resolution of 30 000, measuring masses between 100 and 1700 Da with 1/k_0_ values between 0.6 and 1.6 vs/cm^2^. MS2 scans between 100 and 1700 Da at a resolution of 30 000 were performed on the precursor between charge states of 2–5 with targeted intensities of 20 000, an intensity threshold of 2500 au, and 10 PASEF MS/MS scans were performed in each cycle with cycle times of 1.2 s. The peptides were fragmented using CID with an isolation window of 2 Da and collision energies ranging from 20 eV (1/k_0_ value of 0.6 Vs/cm^2^) to 59 eV (1/k_0_ value of 1.6 Vs/cm^2^). Dynamic exclusion was performed for 30 s.

Data analysis: LC‐MS/MS data were searched against the mouse SwissProtKB database downloaded on 3‐23‐2022 (17 552 entries) using the program MSFragger v3.4. These searches were performed considering full tryptic peptides with no more than 2 missed cleavage sites. The MS1 and MS2 mass accuracies were set to 20 ppm, carbamidomethylation was considered a fixed modification, and oxidation of methionine and protein acetylation were considered variable modifications. The results were filtered using a reverse decoy database strategy with a percolator, and the PSM, peptide, and protein FDR rates were set to 1%. Positively identified proteins were identified by a minimum of two peptides, with at least one of these being unique peptide identifications. Label‐free quantification was performed using the MaxLFQ algorithm. The match between runs was enabled with a mass tolerance of 10 ppm, a retention time shift of 5 min, and a 1/k_0_ tolerance of 0.05. Quantitation was performed on unique peptides; only proteins identified by the two ions were quantified, and the LFQ intensities were normalized to the total peptide content for each LC‐MS experiment. The LFQ intensities were used to determine the abundance ratios of the proteins across groups, and significance was determined using a T‐T‐derived *p*‐value.

### 4.6 Dual‐Flank Xenograft‐Tumor Model and Tissue Processing for Analysis

Two‐month‐old C57BL/6J female mice or female nude mice were challenged with 1×10^6^ MC38 cells suspended in 100 µL PBS in the left flank and modified Tet‐On shKDELR2 MC38 cells in the right flank. Doxycycline was provided in the diet (Bio‐Serv, cat# S3888). B16F10 ffLuc cells were inoculated in a manner similar to that for MC38 cells. Tumor volume was estimated from the length (L) and width (W), based on the formula V  =  1/6π x L x W x W. Mice were terminated when the tumor at either of its dimensions exceeded 2 cm or when tumor volume was larger than 3000 mm^3^. Following euthanasia, the tumors were carefully excised. A portion of each tumor was fixed in 4% paraformaldehyde for 24 h at room temperature and then transferred to ethanol for storage until further processing for histological analysis. The remaining tumor tissue was finely minced using sterile scissors and enzymatically digested in DMEM containing 0.2% collagenase (Sigma‐Aldrich, Cat# SMB0070) at 37 °C with gentle agitation. After digestion, the cell suspension was filtered through a 70 µm cell strainer to remove debris and undigested fragments. The resulting single‐cell suspension was used for downstream flow cytometry analysis.

### 4.7 Immunoblotting

The cells were harvested by centrifugation (4°C, 4000×g for 5 min) and washed with ice‐cold PBS. Cell pellets were lysed in radioimmunoprecipitation assay (RIPA) buffer supplemented with protease inhibitors (Bimake, b14001) and phosphatase inhibitors (Bimake, b15001). Following vortexing for 10 min, the lysates were cleared by centrifugation (4°C, 16 000 × g for 15 min). The supernatant was collected, and the protein content was quantified and mixed with reducing sample buffer, followed by boiling for 5 min. Samples were loaded on GenScript gradient SDS‐PAGE gels, transferred to PVDF membranes (Immobilon‐P) with eBlot (GenScript), detected by chemiluminescence using Immobilon Crescendo (Millipore WBLUR0500), and imaged on a Bio‐Rad ChemiDoc XR. Antibodies that were used are listed here: the anti‐nanoLuc antibody (Promega, cat# N7000, 1:4000) was used according to the manufacturer's instructions. Anti‐α‐tubulin (Cat# 2144S), anti‐PERK (#3192, 1:1000), anti‐ATF4 (#11815, 1:1000), anti‐P‐eIF2α‐S51 (#3398, 1:1000), and anti‐eIF2α (#2103, 1:1000) were purchased from Cell Signaling. Anti‐p97 was used as the loading control (Novus Biologicals, cat# NB100‐1558, 1:4000). HRP‐conjugated goat anti‐mouse and goat anti‐rabbit secondary antibodies were purchased from Jackson ImmunoResearch Laboratories (1:10 000).

### 4.8 Flow Cytometry Analysis

Tumor tissues were mechanically dissociated and passed through 70 µm cell strainers (Stellar Scientific, MD, USA) to obtain single‐cell suspensions. For surface marker staining, the cells were incubated with fluorochrome‐conjugated antibodies for 30 min at room temperature in the dark. The following antibodies were used: PerCP‐conjugated anti‐mouse CD45 (BioLegend, Cat# 103130, 1:50), PerCP‐conjugated anti‐mouse/human CD11b (BioLegend, Cat# 101230, 1:50), PE‐conjugated anti‐mouse CD3ε (STEMCELL Technologies, Cat# 130‐116‐289, 1:50), APC‐eFluor 780‐conjugated anti‐mouse CD4 (eBioscience/Thermo Fisher Scientific, Cat# 47‐0042‐82, 1:50), FITC‐conjugated anti‐mouse CD8a (eBioscience/Thermo Fisher Scientific, Cat# 11‐0081‐82, 1:50), APC‐conjugated anti‐mouse CD44 (Miltenyi Biotec, Cat# 130‐119‐121, 1:50), PerCP‐Vio700‐conjugated anti‐mouse CD62L (Miltenyi Biotec, Cat# 130‐126‐043, 1:100), FITC‐conjugated anti‐human CD68 (BioLegend, Cat# 333805, 1:50), Pacific‐blue conjugated anti‐mouse NK1.1 CD161 (BioLegend, Cat#108721, 1:100), APC‐conjugated anti‐human CD163 (BioLegend, Cat# 326509, 1:50), PE/Cyanine7‐conjugated anti‐human CD64 (BioLegend, Cat# 305021, 1:50), PE‐conjugated anti‐human CD86 (BioLegend Cat# 374203, 1:50), and FITC anti‐mouse Ly6G (BioLegend, Cat#108406, 1:50). Appropriate isotype controls were obtained from BioLegend and BD Biosciences. We also used the anti‐mouse CD16/CD32 (Mouse BD Fc block, Cat#553142, BD Biosciences, USA) before staining with any specific antibodies for the evaluation of TME.

For intracellular staining, the cells were fixed and permeabilized using a BD Cytofix/Cytoperm Fixation/Permeabilization Kit (BD Biosciences, Cat# 554714, 1:50) followed by staining with Alexa Fluor 488‐conjugated anti‐iNOS (Thermo Fisher Scientific, Cat# 53‐5920‐82, 1:100), APC‐conjugated anti‐Arginase‐1 (Arg1) (Thermo Fisher Scientific, Cat# 17‐3697‐82, 1:100), PE anti‐mouse Arginase 1 (BioLegend, Cat# 165804, 1:100), and Brilliant Violet 711 anti‐mouse CD54 (BioLegend, Cat# 116143, 1:100).

Stained cells were acquired on a CytoFLEX S flow cytometer (Beckman Coulter) and analyzed using the CytExpert software. Gating strategies are provided in the supplemental supporting data (Figures S10 and S11). Appropriate fluorescence minus one (FMO) controls were used to guide gating and ensure accurate identification of cell populations.

### 4.9 Immunohistochemistry and Immunofluorescence for Macrophage, Neutrophil, and MCP1/CCL2 Analysis

Tumor tissues were fixed in 4% paraformaldehyde, dehydrated through an ethanol gradient, embedded in paraffin wax, and sectioned at 5 µm thickness. Paraffin‐embedded sections were deparaffinized in xylene, rehydrated using descending ethanol concentrations, and rinsed in phosphate‐buffered saline (PBS). Antigen retrieval was performed by boiling the sections in 10 mM sodium citrate buffer (pH 6.0) for 10 min.

After cooling, the sections were blocked for 1 h at room temperature in PBS containing 1% bovine serum albumin (BSA) and 2% goat serum. Slides were then incubated overnight at 4°C with primary antibodies: rat anti‐CD68 (Novus Biologicals, NBP2‐33337SS, 1:100) and anti‐MCP1/CCL2 (Thermo Fisher Scientific, Cat# MA5‐17040, 1:200).

For immunofluorescence, the slides were washed three times in PBS with 0.1% Tween‐20 (PBST), followed by 1 h incubation at room temperature with fluorophore‐conjugated secondary antibodies: Alexa Fluor 488 (Jackson ImmunoResearch, Cat# 115‐545‐003, 1:500) and Alexa Fluor 546 (Thermo Fisher Scientific, Cat# A‐11081, 1:500). After the final washes, sections were mounted using VECTASHIELD PLUS Antifade Mounting Medium with DAPI (Vector Laboratories, Cat# H‐2000). Imaging was performed using a Leica SP8 confocal microscope (Leica Microsystems), and images were analyzed using the LAS X software (Leica Application Suite X).

For chromogenic immunohistochemistry (IHC), adjacent sections were similarly processed for antigen retrieval and primary antibody incubation. After washing, the sections were incubated with HRP‐conjugated secondary antibodies and developed using 3,3'‐diaminobenzidine (DAB) substrate. Nuclei were counterstained with hematoxylin. The slides were visualized using an Olympus microscope equipped with differential interference contrast (DIC) optics (Olympus).

### 4.10 Generation of BMDMs, Polarization Assays, and Chemotactic Response

BMDMs were generated from femur bone marrow cells of 2‐month‐old female C57BL/6J mice. In brief, after flushing the bone marrow cells using a syringe and 23G needle, red blood cells were removed with ACK lysis buffer (Cat# A1049201, Thermo Fisher, USA). After washing, the cells were cultured in a bone marrow culture medium (DMEM supplemented with 10% FBS, 1% penicillin‐streptomycin, and 1 ng/mL M‐CSF1) for six days. On day 7, BMDMs were washed twice with DMEM and treated with 30% v/v concentrated supernatants collected from shKDELR2‐MC38 cells diluted in DMEM for 48 h. For positive controls, M1 polarization was induced using IFNγ (20 ng/mL), PeproTech, Thermo Fisher, cat#AF‐315‐05‐20UG, and LPS (240 ng/mL, Sigma‐Aldrich, Cat#SMB00704), while M2 polarization was induced using IL‐4 (20 ng/mL, PeproTech, Thermo Fisher, Cat#214‐14‐20UG). The cells were analyzed by flow cytometry. M1 macrophages were identified as double‐positive for anti‐F4/80 and anti‐iNOS staining, whereas M2 macrophages were identified as double‐positive for anti‐F4/80 and anti‐Arginase 1 staining.

In a separate experiment, chemotaxis was evaluated using the Cell Migration/Chemotaxis Assay Kit from Abcam (Cat# ab235673) following the manufacturer's instructions. Briefly, BMDMs were seeded in the upper chamber of the assay insert, which contained a porous membrane. The lower chamber was filled with a supernatant of shKDELR2‐MC38 cells. The cells were incubated for 24 h. A positive control for the chemotaxis assay was included, as described in the kit protocol.

### 4.11 Generation of Human Naïve Macrophages

Human samples used in this study were obtained from healthy donors under an Institutional Review Board (IRB)‐approved protocol at University Hospitals Cleveland Medical Center (04‐04‐22). Samples were de‐identified prior to use. Apheresis cells of a healthy donor were provided from the Hematopoietic Biorepository blood bank of the Case Comprehensive Cancer Center under consent, affirming that the experiments conformed to the principles set out in the WMA Declaration of Helsinki and the Department of Health and Human Services Belmont Report. Macrophages were generated according to [70]. In brief, cells were gently layered over a Ficoll gradient (density 1.077 g/mL) in 50 mL conical tubes. Samples were centrifuged at 1000 × g for 20 min at room temperature with the brake disengaged. The PBMC layer was collected, washed twice in PBS containing 0.5% bovine serum albumin (BSA), and pelleted at 400 × g for 10 min. Cell number and viability were determined using trypan blue exclusion. 10^7^ cells were seeded in 10 cm plates in RPMI‐1640 medium supplemented with 10% fetal bovine serum (FBS) and incubated for 1.5 h at 37°C, and 5% CO_2_ to allow monocyte adherence. Non‐adherent lymphocytes were removed by washing with PBS, and adherent monocytes were differentiated into macrophages by culturing for four days in RPMI‐1640 containing 10% FBS and 100 ng/mL recombinant human macrophage colony‐stimulating factor (M‐CSF), with medium replaced after 2 days. For polarization positive controls, macrophages were stimulated for 2 days with either 20 ng/mL lipopolysaccharide (LPS) and 90 ng/mL IFN‐γ to generate M1 differentiation or 25 ng/mL IL‐4 to generate M2 macrophages. For polarization analysis, HCC1806 cells were seeded at 2 × 10^5^ cells per well in a 6‐well plate and treated with 10 µL/mL LNP–siKDELR2 or LNP–scramble for 24 h. After treatment, the cells were washed with fresh medium and cultured for an additional 2 days. The resulting conditioned medium was collected and added to naive macrophages at a density of 1 × 10^6^ cells per well in a 6‐well plate. Macrophages were incubated with this conditioned medium for 2 days to induce polarization before flow cytometry analysis.

### 4.12 Immunodepletion of KDEL Proteins From Tumor Cell Supernatants and Functional Analysis

The culture supernatants from both MC38 and shKDELR2‐MC38 cells were incubated overnight at 4 °C with either anti‐KDEL (Invitrogen, Thermo Fisher, cat# MA5‐27581) or anti‐GFP antibodies (Invitrogen, Thermo Fisher, cat# MA5‐15256) bound to Protein A/G agarose beads (2 µg antibody per 20–30 µL beads for every 1 mL of supernatant). After incubation, the samples were centrifuged at 500 × g for 5 min at 4°C to pellet the beads. The resulting supernatants were collected, passed through a 0.22 µm filter, and added to day 6 BMDM cultures for 2 days of incubation. Following treatment, the cells were harvested and analyzed using flow cytometry. M1 macrophages were identified based on dual staining for F4/80 and iNOS, and their frequencies were compared to those of positive controls that had been treated with non‐depleted (anti‐GFP) supernatants.

### 4.13 Phagocytosis Assay Using IFNγ/LPS‐Activated BMDMs Co‐Cultured With CFSE‐Labeled MC38 or shKDELR2‐MC38 Cells

As outlined above, BMDMs were cultured in a bone marrow culture medium for six days. On day 7, IFN‐γ (20 ng/mL) and LPS (240 ng/mL) were added to induce activation. After 48 h of stimulation, the cells were harvested and transferred to serum‐starved medium for 12 h for phagocytosis assay.

Separately, 2 × 10^6^ MC38 or shKDELR2‐MC38 knockout cells were harvested and labeled with CFSE (5 µM) for 10 min. Following BMDM harvesting, 2 × 10^4^ macrophages were co‐cultured with equal numbers of CFSE‐labeled MC38 or shKDELR2‐MC38 cells for 2 h. After co‐culture, cells were collected in FACS tubes, stained with anti‐mouse F4/80 antibody, and analyzed for phagocytosis efficiency. Cells that were double positive for CFSE and F4/80 were sorted using a BD Aria III cell sorter and further examined using a Leica SP8 confocal microscope.

### 4.14 Phagocytosis Assay Using IFNγ/LPS‐Activated Human Macrophages Co‐Cultured With CellTrace‐Labeled HCC1806 Cells

M1 macrophages were replated in a 6‐well plate at 2 × 10^5^ cells per well and allowed to adhere overnight in RPMI‐1640 with 10% FBS. HCC1806 was treated with lipid nanoparticles (LNPs) containing either siRNA targeting KDELR2 or a non‐targeting control (10 µL/mL) for 24 h.

After treatment, tumor cells were labeled with CellTrace Far Red, washed with Hank's balanced salt solution (HBSS), and co‐cultured with macrophages at a 1:1 effector‐to‐target ratio (2 × 10^5^ macrophages: 2 × 10^5^ tumor cells per well) for 18 h at 37°C and 5% CO_2_. Following co‐culture, adherent and non‐adherent cells were collected by gentle trypsinization, pooled, and washed with PBS containing 2% FBS and 2 mM EDTA. Cells were stained with FITC anti‐human CD68 antibody for 15 min on ice, washed, and analyzed by flow cytometry. Phagocytic activity was quantified as the proportion of CD68^+^ macrophages positive for CellTrace Far Red fluorescence.

### 4.15 Cytokine Antibody Array Analysis

Cytokine profiling was performed using the mouse cytokine antibody array membrane kit (Abcam ab133995, USA) according to the manufacturer's instructions. Briefly, conditioned media samples (KDELR2 suppression/control) were incubated on the BMDM for 24 h and then collected for the cytokine analysis in vitro. For the in vivo analysis, the tumor lysates at the end of the experimental period (after LNP injection) were prepared under defined conditions as per the manufacturer's instructions to minimize background interference. An equal amount of protein was incubated overnight with array membranes pre‐spotted with capture antibodies against 62 different cytokines. Following incubation, membranes were washed and incubated with a cocktail of biotinylated detection antibodies, followed by HRP‐conjugated streptavidin. Signal detection was carried out using chemiluminescence, and membranes were imaged using the Biofuraw chemi Dog 5200T system (Biofuran, Woburn, MA, USA). For quantitative analysis, spot intensities were measured using ImageJ software (version 1.54j, NIH, USA). Background signal was subtracted from each spot, and values were normalized to internal positive control spots present on each membrane. For heat map visualization, normalized values were further transformed into row‐wise Z‐scores to allow comparison across cytokines.

### 4.16 Immune Memory Assessment in MC38‐ffLuc Re‐Challenge Model

C57BL/6 mice were subcutaneously implanted with a mixture of Tet‐on‐shKDELR2 MC38 cells and parental MC38 cells at a 10:1 ratio, and a separate group with Tet‐on‐shKDELR2 MC38. Upon tumor establishment, animals were maintained on a DOX‐supplemented diet for two weeks to induce shRNA expression. The tumor volumes were monitored weekly using calipers. Once complete tumor regression was observed (volume approaching zero), DOX administration was discontinued by switching the mice to normal chow for a period of 1 week. A group of animals was sacrificed, and spleens were analyzed for markers of T cell memory. The rest were re‐challenged with MC38 cells stably expressing firefly luciferase (MC38‐ffLuc) via subcutaneous injection. A separate cohort of naïve C57BL/6 mice that received the same Luc‐labeled MC38‐ffLuc cells served as the control group. Tumor progression in both the experimental and control groups was assessed longitudinally using bioluminescence imaging (BLI) performed on the IVIS Spectrum system (PerkinElmer, USA).

Prior to imaging, the mice were injected intraperitoneally with 100 mg/kg D‐luciferin. Anesthesia was induced and maintained using vaporized isoflurane (Abbott Laboratories, IL, USA), and the mice were positioned in the imaging chamber. Bioluminescent signals were captured 8 min post‐injection, with a fixed exposure time of 60 s per mouse, throughout the duration of the study.

Quantitative analysis of the bioluminescence data was conducted using the Living Image software platform (PerkinElmer). Photon flux was quantified as the average radiance (photons/s/cm^2^/sr) within a defined rectangular region of interest (ROI) encompassing the tumor‐bearing area. Raw data were further processed and visualized using Newton module integration within the Amira platform and MATLAB (MathWorks, USA) to ensure consistency in image registration and signal normalization across the time points.

### 4.17 Adoptive Transfer of OT‐I T Cells

Naïve T cells were isolated from the spleen of OT‐I mice using the EasySep Mouse T Cell Isolation Kit (Catalog # 19851). The cells were labeled with CFSE (Thermo Fisher, cat# C34554) according to the manufacturer's instructions. A total of 10^7^ cells were suspended in 200 µL PBS and injected intravenously into tumor‐bearing C57BL/6 mice.

### 4.18 Formulation of siRNA LNPs and Their Biophysical Characterization by Gel Retardation and DLS

ECO (MW ¼ 1023) at a stock concentration of 50 mM in ethanol was vortexed with siScramble duplex: sense 5′‐GUUUCACACGUCGUACUCACU‐3′ and antisense 5′‐AGUGAGUACGACGUGUGAAAC‐3,’ siKDELR2 sense: 5′‐GACCAUUCUCUACUGCGACUU‐3′; antisense: 5′‐AAGUCGCAGUAGAGAAUGGUC‐3’ at a stock concentration of 250 µM in nuclease‐free water for 30 min to form nanoparticles at N/P = 8 with a final siRNA concentration of 100 nM. Helper lipids, beta‐sitosterol (BCHOL) (Millipore Sigma), were added by combining an equivalent volume of a helper lipid with ECO at a concentration of 0.5 mM. Agarose gels (1% w/v) were prepared in Tris/borate/ethylenediaminetetraacetic acid (EDTA) buffer with ethidium bromide added for the gel retardation assay. A nanoparticle formulation (10 µL) was added to 2 µL of DNA gel‐loading dye (6X, Life Technologies) and ran at 100 V for 20 min. The gel was imaged using a ChemiDoc XRS system (Bio‐Rad). The nanoparticle size and zeta potential were measured using dynamic light scattering on an Anton Paar Litesizer.

For human KDELR2 we used a combination of two siRNA sequences: sequence 1 sense: 5′‐ACACAUCUAUGAAGGUUAUCU‐3′; antisense: 5′‐ AUAACCUUCAUAGAUGUGUUA‐3’; sequence 2: sense: 5′‐ GAUCUGGCGCUUCUACUUUGA‐3′; antisense: 5′‐ AAAGUAGAAGCGCCAGAUCCA‐3’.

### 4.19 LNP Treatment in MC38‐ffLuc and B16F10‐ffLuc Xenograft Models

When tumors derived from MC38‐ffLuc or B16F10‐ffLuc cells reached an average volume of ∼160 mm^3^, a single intratumoral injection of lipid nanoparticles (LNPs) containing either scrambled siRNA (control) or siRNA targeting KDELR2 (siKDELR2) was administered in a fixed volume of 60 µL. Tumor progression was subsequently monitored using bioluminescence imaging (BLI) to evaluate changes in tumor burden over time.

Imaging was continued until a marked reduction in luminescent signal was observed in the siKDELR2‐treated group relative to the scramble control group, and until tumors in the control group reached volumes near the ethical endpoint (necrosis or ulceration).

In parallel, two additional cohorts for each tumor model underwent the same LNP treatment regimen. However, in these groups, tumors were harvested 2 weeks post‐injection for immunological characterization, including analyses of tumor‐infiltrating immune cells and cytokine profiling within the TME. For systemic delivery studies, mice received intravenous injections of LNP‐formulated siRNAs via the tail vein on days 8, 10, and 12 following tumor implantations. Tumor growth and immune profiling were performed as described for intratumor administration.

### 4.20 Orthotopic Implantation and LNP Treatment in E0771‐ffLuc Mammary Tumor Model

E0771‐ffLuc cells were cultured under standard conditions and resuspended at a concentration of 5 × 10^6^ cells/mL in sterile PBS. To preserve the cell viability, the cell suspension was kept on ice and used for implantation within 1 h of preparation. For orthotopic tumor establishment, 100 µL of the cell suspension was injected into the mammary fat pad between the second and third mammary glands of female C57BL/6 mice using a 28.5‐gauge needle.

Following tumor engraftment, bioluminescence imaging (BLI) was performed to monitor tumor development. Once the tumors reached a palpable volume of approximately 180 mm^3^, a single intratumoral injection of lipid nanoparticles (LNPs) containing either scrambled siRNA (control) or siKDELR2 was administered at a volume of 60 µL per tumor.

Tumor progression was monitored longitudinally via BLI to assess changes in the luminescence signal intensity, reflecting the treatment response. At the end of the study, tumors were harvested for comprehensive immunological analyses to evaluate the TME, including immune cell infiltration and cytokine expression profiles.

### 4.21 Statistical Analysis

No animal, sample, or data were excluded from the analysis. Experimental animal groups were assigned randomly to vehicle or treatment groups. No blinding was performed. The number of samples included in each experiment is indicated in figure legends. Statistical analyses were performed using R or GraphPad Prism software, version 11.0.1(99). The values are reported as the mean ± SD. Data were compared between groups using one‐way analysis of variance (ANOVA) followed by Welch's *t*‐test. Comparisons between the two groups were performed using Welch's unpaired *t*‐test. *P* was set at *p* < 0.05. In vivo experiments were performed in a semi‐blind fashion.

## Author Contributions

**Zheng‐Rong Lu**: conceptualization, methodology. **Boaz Tirosh**: conceptualization, supervision, resources, project administration, writing – review and editing, writing – original draft, formal analysis, funding acquisition. **Hong Wang**: methodology. **Belinda Willard**: data curation, validation. **Timothy A. Chan**: writing – original draft, writing – review and editing.

## Conflicts of Interest

The authors declare no conflicts of interest.

## Supporting information




**Supporting File**: advs76148‐sup‐0001‐suppMat.pdf.

## Data Availability

Supporting data related to this study are included in the supplementary files. All other data that support the findings of this study are available from the corresponding author upon reasonable request. The data that support the findings of this study are openly available in PRIDE at https://www.ebi.ac.uk/pride/archive/projects/PXD072994, reference number PXD072994.
