# Supplementary material for: Inhibition of KDEL Receptors Remodels the Tumor Microenvironment for T Cell Independent Tumor Regression
Source: Adv Sci (Weinh). 2026 Jun 23:e76148. Online ahead of print. doi: 10.1002/advs.76148 (PMC13335452; doi:10.1002/advs.76148)
Supplement: Supplementary file 1 — Supporting File: advs76148‐sup‐0001‐suppMat.pdf. [file ADVS-9999-e76148-s001.pdf]

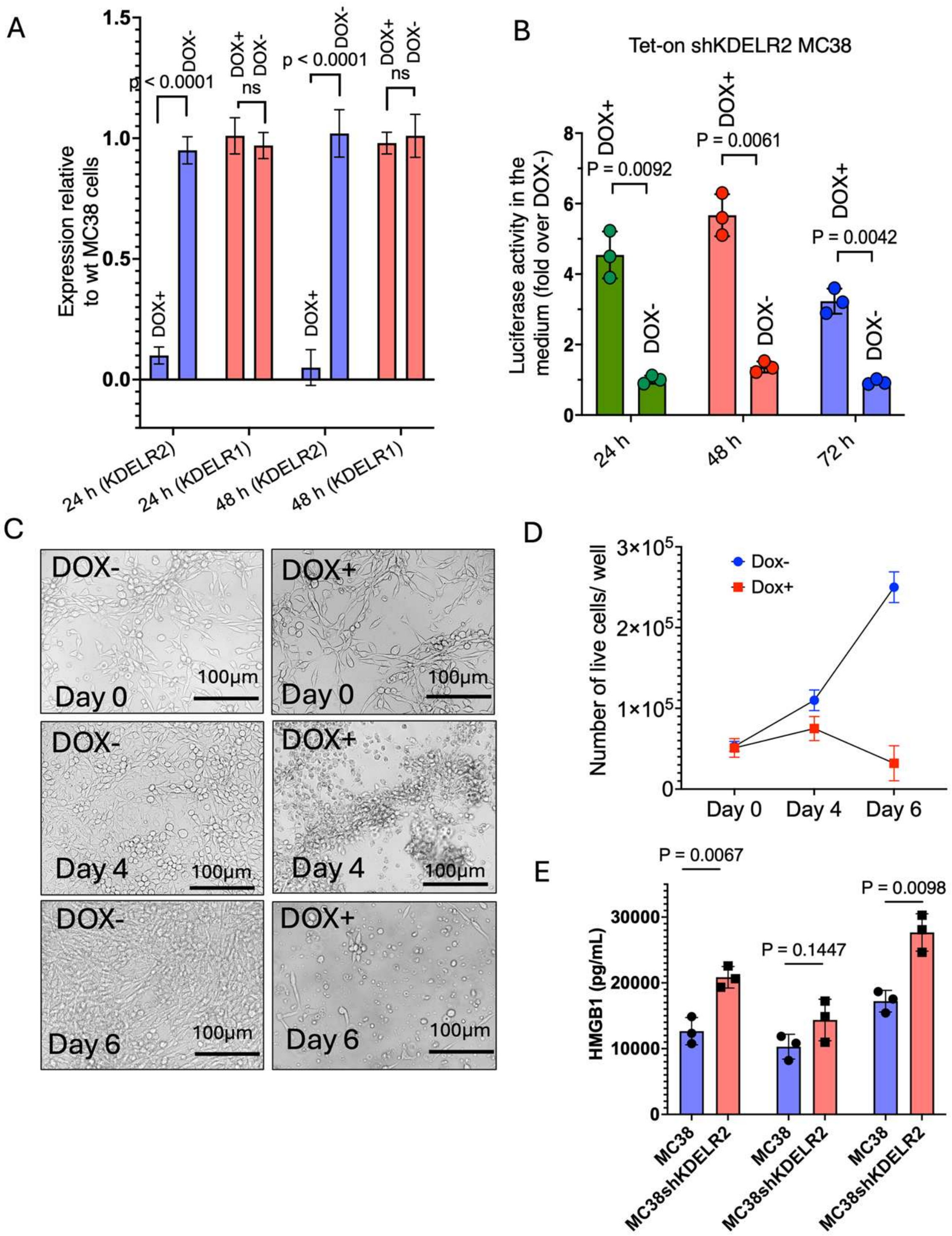

Figure S1: Time-dependent validation of KDELR2 silencing and stability of KDELR1 expression with sustained induction of KDEL-protein secretion, and Effect of KDELR2 inhibition on cell viability. A. qPCR analysis of KDELR1 expression in Tet-On shKDELR2 MC38 cells following doxycycline (DOX) induction at 24 and 48 hours. KDELR1 expression remains unchanged upon KDELR2 knockdown, indicating a lack of compensatory upregulation. Data are presented as mean ± SD (N = independent replicates). Statistical significance was determined using Welch's unpaired t-test; ns, not significant. **B.** Quantification of secreted KDEL-reporter activity in culture supernatants at the indicated time points following DOX induction. KDELR2 silencing results in a significant and sustained increase in extracellular reporter activity at 24 and 48 hours compared to DOX− controls. **C.** MC38 Tet-On shKDELR2 were treated with or without 10 µM of doxycycline (DOX). Cells were imaged under a light microscope. **D.** Live cells were counted and plotted over time (N=3, mean ± SD). **E.** MC38 Tet-On shKDELR2 were treated with or without 10 µM of doxycycline (DOX) for 24 h. Supernatants were analyzed for HMGB1 content by ELISA. Shown are three individual experiments, each one performed in triplicate (N=3, mean ± SD). Statistical significance was determined using Welch's *t*-test; $p < 0.05$ was considered significant.

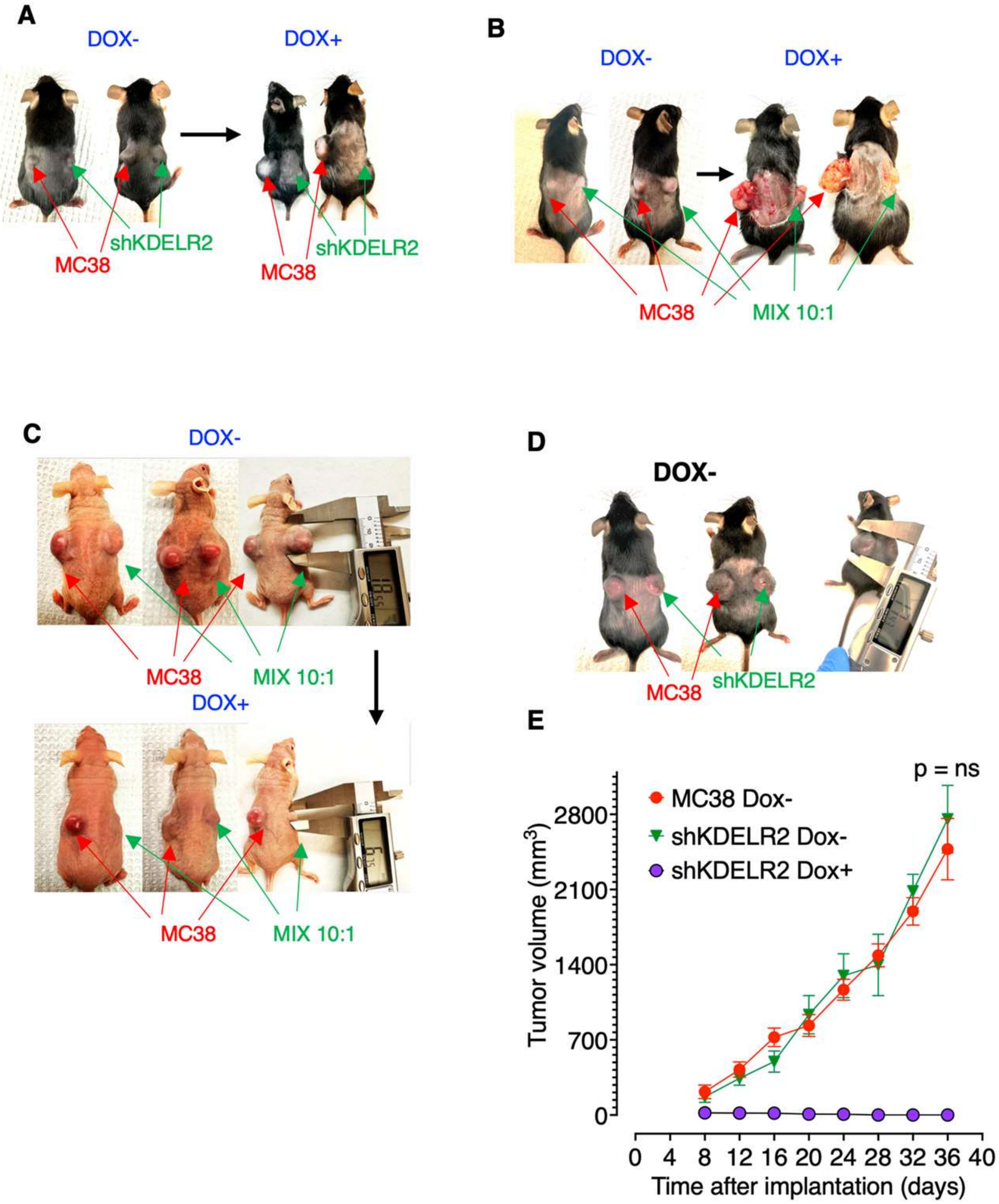

Figure S2: In the absence of DOX, MC38 and Tet-On shKDELR2 cells have equivalent tumorigenicity. **A.** Representative images showing comparable tumor size of MC38 and Tet-On shKDELR2 cells when grown in mice fed by normal chow. **B.** Tumor volume measurements over time demonstrate similar growth patterns of MC38 wt and Tet-On shKDELR2 cells in C57BL/6 mice fed on normal chow. When mice were fed with a DOX diet, Tet-On shKDELR2 did not develop tumors (N = 3, mean ± SD, for each cohort). Statistical significance was determined using Welch's *t*-test; $p < 0.05$ was considered significant. **C.** Representative images of mice challenged with MC38 and a MIX 10:1 between wt MC38 and Tet-On shKDELR2 cells, when grown in C57BL/6 mice fed by chow supplemented or not with DOX. **D.** Representative images of mice challenged with MC38 and a MIX 10:1 between wt MC38 and Tet-On shKDELR2 cells, when grown in nude mice fed by chow supplemented or not with DOX.

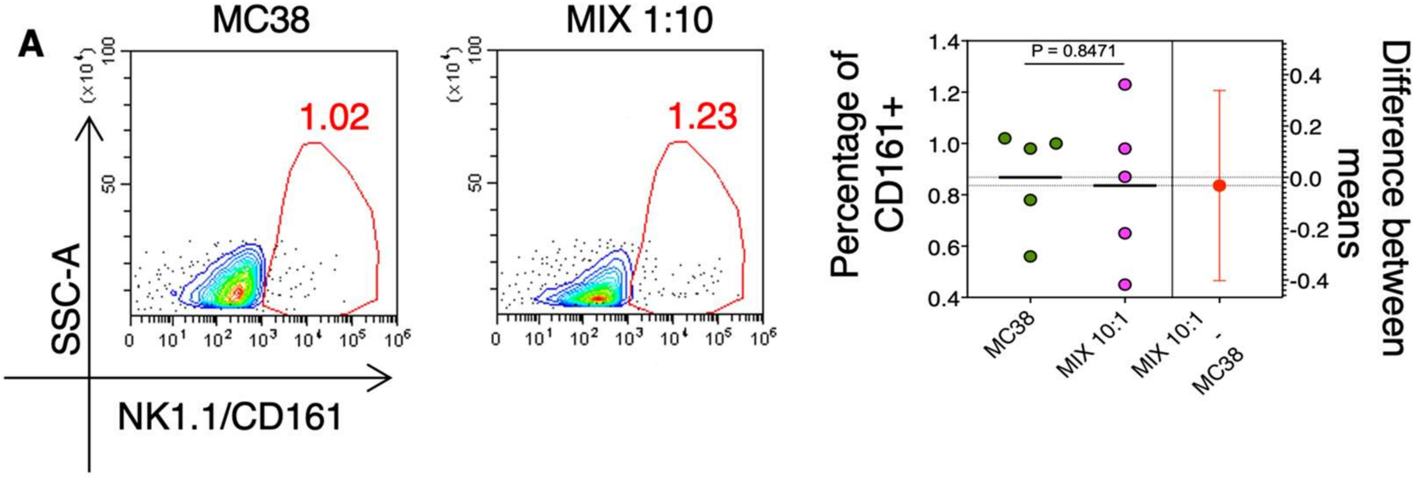

Figure S3: NK cells are not enriched in the TME after silencing of shKDELR2. Analysis of MC38 tumors for infiltration of NK cells. MC38 (WT and Tet-On shKEDLR2) were allowed to grow. Two weeks after placing the mice on a DOX diet, tumors were isolated, dissociated into single cells and analyzed for $CD161^{+}$ NK1.1 cells. No significant infiltration of $CD161^{+}$ NK1.1 cells was observed for MIX 10:1 and MC38 groups in C57BL/6 hosts (N = 3). Statistical significance was determined using Welch's unpaired *t*-test; $p < 0.05$ was considered significant.

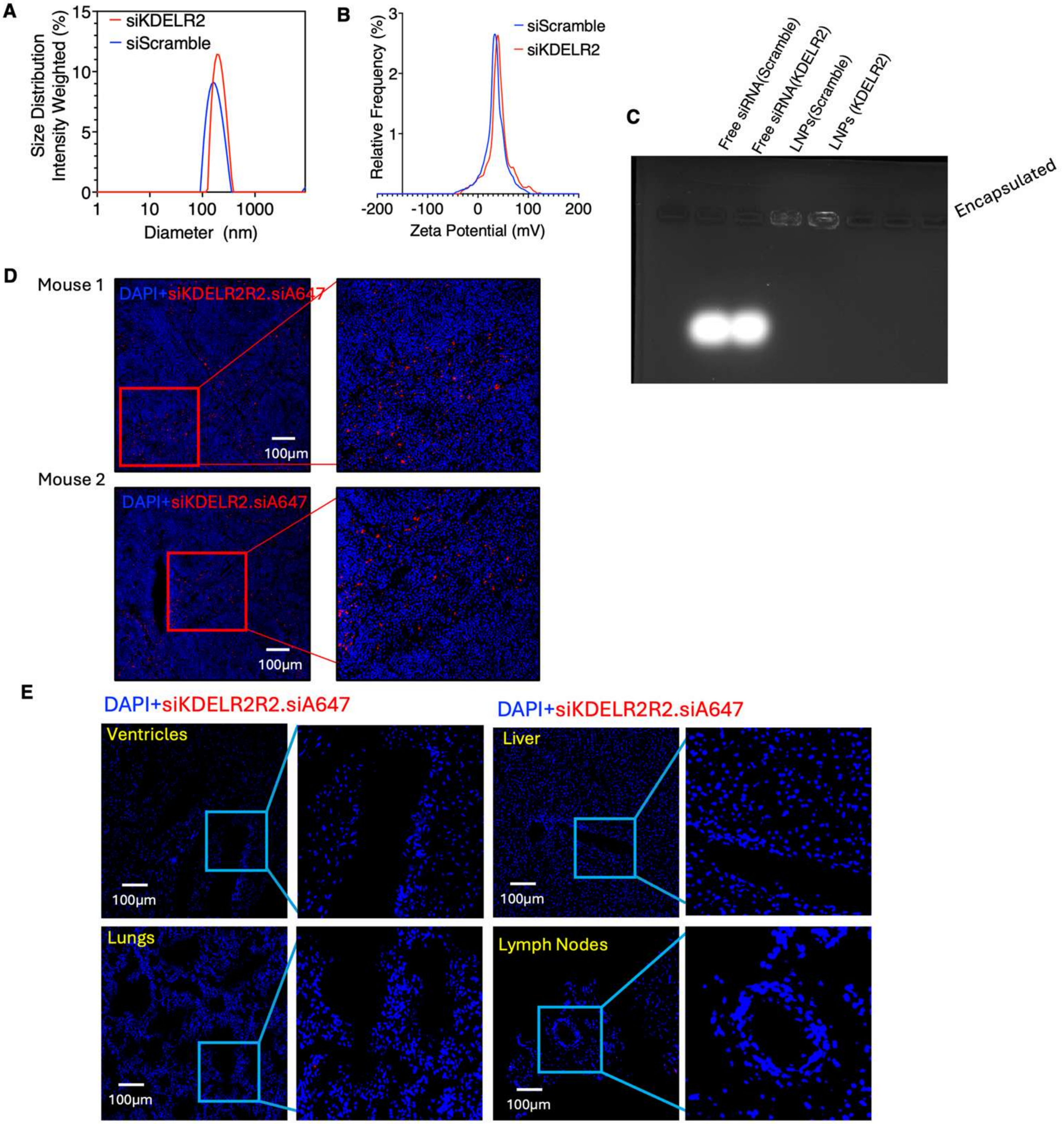

Figure S4: Characterization of Lipid Nanoparticles (LNPs) encapsulating scramble and siKDELR2 siRNA and the biodistribution following a single intratumoral injection. A. Dynamic light scattering (DLS) was used to assess the particle size distribution of the respective LNP formulations. **B.** Zeta potential measurements were performed to evaluate the surface charge of LNPs formulated with scramble and siKDELR2 siRNA. **C.** Agarose gel electrophoresis was conducted to assess siRNA encapsulation efficiency within the LNPs, demonstrating complete encapsulation of siRNA in both formulations. **D.** MC38 tumors of 300-500 mm^3 were injected with 70 µl of Alexa647-labeled LNPs. Four days later, mice were sacrificed, and tumor tissue was analyzed by fluorescent microscopy for the presence of LNPs. Shown are two representative tumor images. **E.** Distant organs were isolated and analyzed. LNPs were not observed in any.

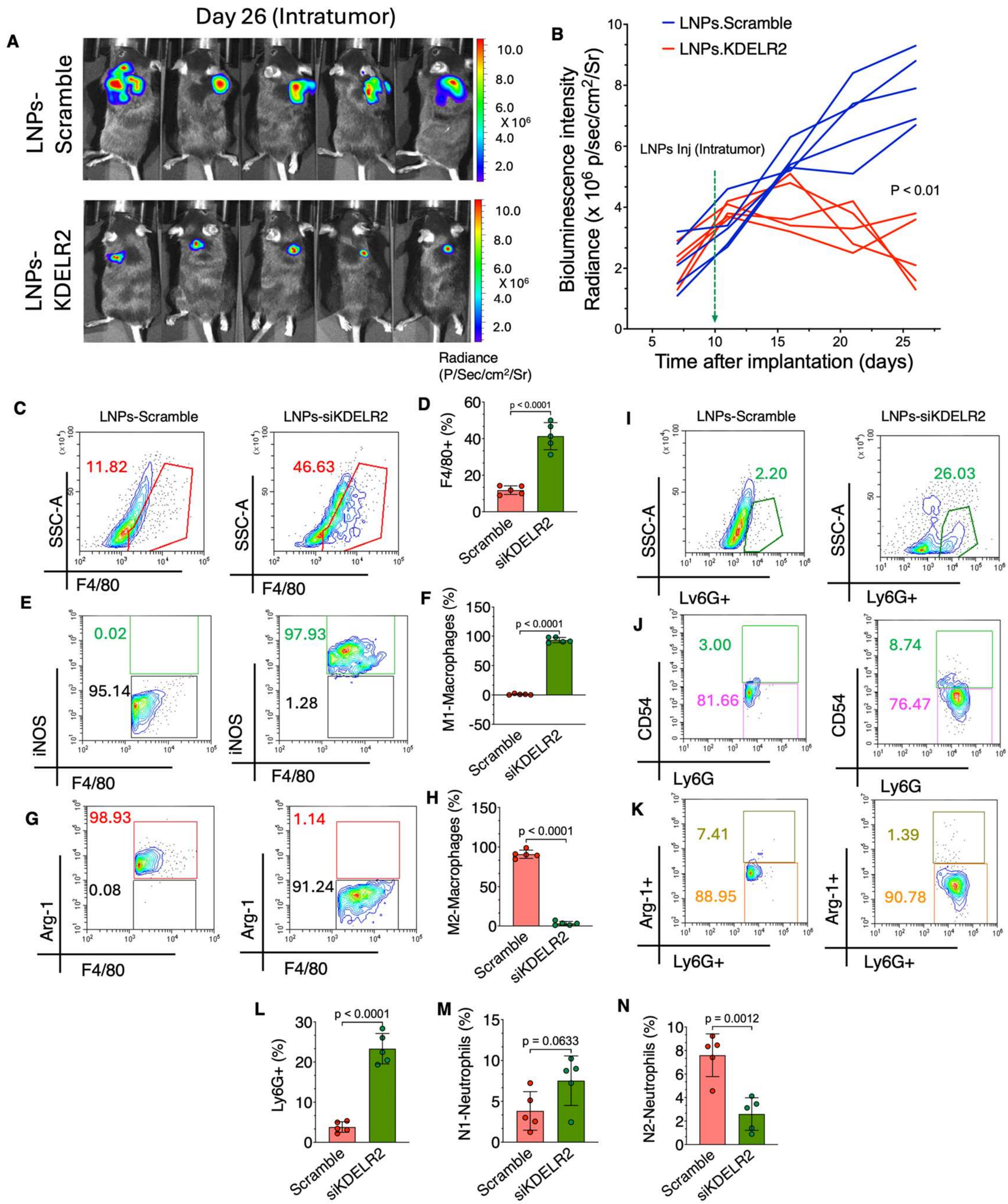

A
Day 26 (Intratumor)
LNPs-Scramble
LNPs-KDELR2
Radiance (P/Sec/cm²/Sr)
X 10⁶
B
LNPs.Scramble
LNPs.KDELR2
LNPs Inj (Intratumor)
P < 0.01
Bioluminescence intensity Radiance (x 10⁶ p/sec/cm²/Sr)
Time after implantation (days)
C
LNPs-Scramble
LNPs-siKDELR2
11.82
46.63
SSC-A
F4/80
D
p < 0.0001
F4/80+ (%)
Scramble
siKDELR2
E
0.02
95.14
97.93
1.28
iNOS
F
M1-Macrophages (%)
G
98.93
0.08
1.14
91.24
Arg-1
H
M2-Macrophages (%)
I
2.20
26.03
Ly6G+
J
3.00
81.66
8.74
76.47
CD54
Ly6G
K
7.41
88.95
1.39
90.78
Arg-1+
L
Ly6G+ (%)
M
p = 0.0633
N1-Neutrophils (%)
N
p = 0.0012
N2-Neutrophils (%)

Figure S5: KDELR2 inhibition induces macrophage-dominant immune remodeling and non-suppressive neutrophil infiltration in tumors: A. Representative bioluminescence imaging of tumor-bearing mice treated with intratumoral injection of LNPs carrying scrambled siRNA or siKDELR2 at day 26 post-implantation. **B.** Tumor growth kinetics measured as luminescence radiance over time following intratumoral LNP injection (indicated by arrow). KDELR2 silencing significantly suppresses tumor progression compared to control ($P < 0.01$). **C–D.** Flow cytometry analysis and quantification of tumor-infiltrating macrophages (F4/80$^+$), showing increased macrophage infiltration upon KDELR2 silencing. **E–F.** Macrophage polarization analysis demonstrating increased M1 macrophages (iNOS$^+$ F4/80$^+$) in siKDELR2-treated tumors. **G–H.** Analysis of M2 macrophages (Arg-1$^+$ F4/80$^+$) showing a marked reduction following KDELR2 silencing. **I–J.** Flow cytometry analysis of tumor-infiltrating neutrophils (Ly6G$^+$) and CD54 expression, indicating enhanced recruitment and partial activation of neutrophils. **K.** Arginase-1 expression in Ly6G$^+$ neutrophils, demonstrating low levels of immunosuppressive phenotype. **L.** Quantification of total Ly6G$^+$ neutrophils showing increased infiltration in siKDELR2-treated tumors. **M–N.** Quantification of neutrophil subsets, indicating modest increase in CD54$^+$ (N1-like) neutrophils and reduction of Arg-1$^+$ (N2-like) neutrophils, N = 5; Statistical significance was determined using Welch's unpaired *t*-test; $p < 0.05$ was considered significant.

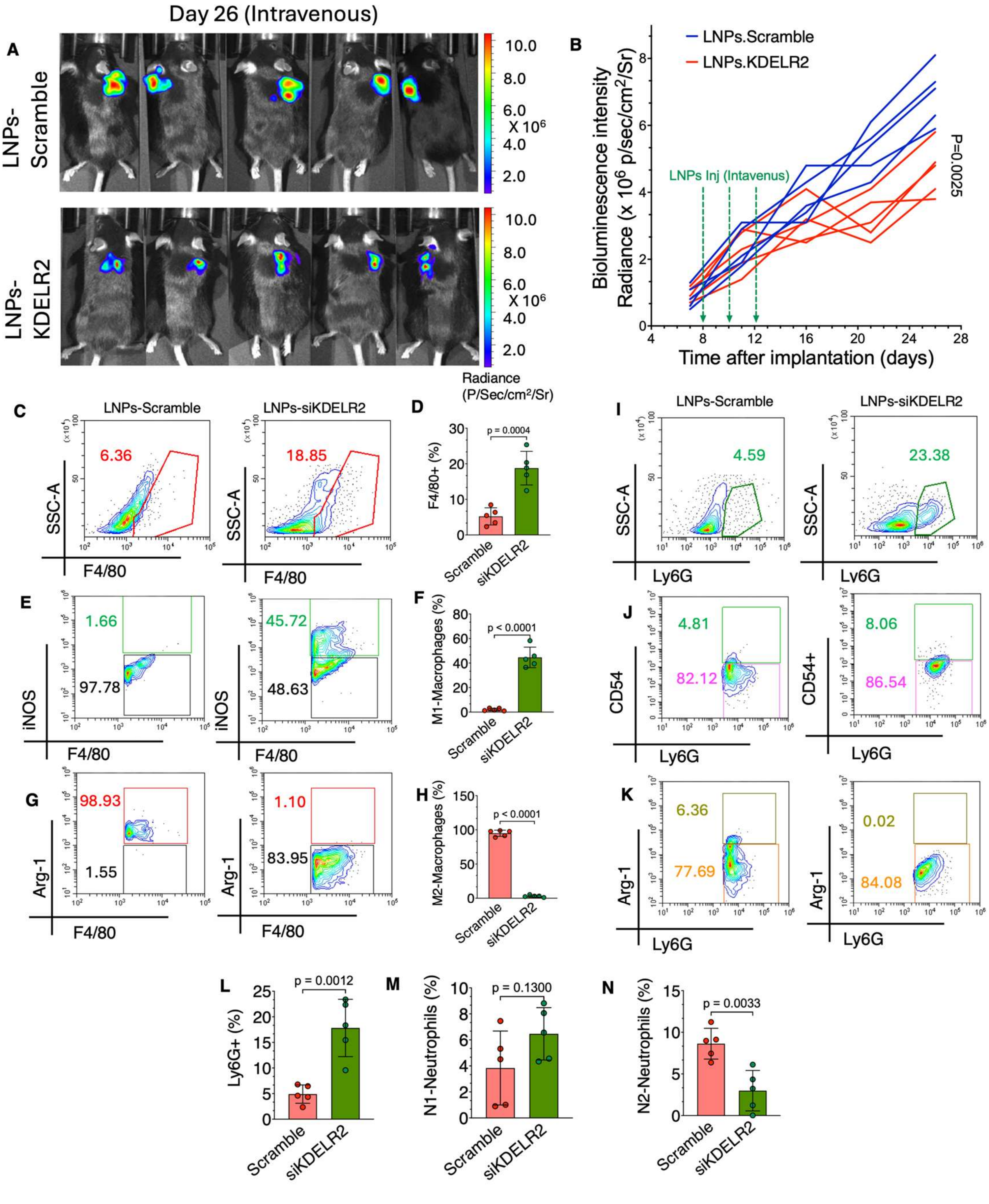
Day 26 (Intravenous)
A
LNPs-Scramble
LNPs-KDELR2
10.0
8.0
6.0
X 10^6
4.0
2.0
Radiance (P/Sec/cm²/Sr)
B
LNPs.Scramble
LNPs.KDELR2
LNPs Inj (Intavenus)
P=0.0025
Bioluminescence intensity
Radiance (x 10^6 p/sec/cm²/Sr)
Time after implantation (days)
C
LNPs-Scramble
LNPs-siKDELR2
6.36
18.85
SSC-A
F4/80
D
F4/80+ (%)
p = 0.0004
Scramble
siKDELR2
E
1.66
97.78
45.72
48.63
iNOS
F
M1-Macrophages (%)
p < 0.0001
G
98.93
1.55
1.10
83.95
Arg-1
H
M2-Macrophages (%)
p < 0.0001
I
LNPs-Scramble
LNPs-siKDELR2
4.59
23.38
Ly6G
J
4.81
82.12
8.06
86.54
CD54
CD54+
K
6.36
77.69
0.02
84.08
L
Ly6G+ (%)
p = 0.0012
M
N1-Neutrophils (%)
p = 0.1300
N
N2-Neutrophils (%)
p = 0.0033

Figure S6: Systemic delivery of LNP-siKDELR2 induces partial tumor suppression and immune remodeling in the tumor microenvironment. A. Representative bioluminescence imaging of tumor-bearing mice following intravenous administration of LNPs carrying scrambled siRNA or siKDELR2 at day 26 post-implantation. **B.** Tumor growth kinetics measured as luminescence radiance over time following intravenous LNP injections (indicated by arrows). KDELR2 silencing results in a modest but significant reduction in tumor progression compared to control ($P < 0.05$). **C–D.** Flow cytometry analysis and quantification of tumor-infiltrating macrophages (F4/80$^+$), demonstrating increased macrophage infiltration following systemic siKDELR2 delivery. **E–F.** Analysis of macrophage polarization showing increased M1 macrophages (iNOS$^+$ F4/80$^+$) in siKDELR2-treated tumors. **G–H.** Analysis of M2 macrophages (Arg-1$^+$ F4/80$^+$), showing a marked reduction upon KDELR2 silencing. **I–J.** Flow cytometry analysis of Ly6G$^+$ neutrophils and CD54 expression, indicating increased neutrophil recruitment and partial activation following treatment. **K.** Arginase-1 expression in Ly6G$^+$ neutrophils, demonstrating low levels of immunosuppressive phenotype. **L.** Quantification of total Ly6G$^+$ neutrophils showing increased infiltration in siKDELR2-treated tumors. **M–N.** Quantification of neutrophil subsets, showing a non-significant trend toward increased CD54$^+$ (N1-like) neutrophils and a significant reduction in Arg-1$^+$ (N2-like) neutrophils. Overall, systemic KDELR2 silencing induces macrophage-dominant immune remodeling and recruitment of predominantly non-suppressive neutrophils, although with reduced efficacy compared to intratumoral delivery, N = 5; Statistical significance was determined using Welch's unpaired *t*-test; $p < 0.05$ was considered significant.

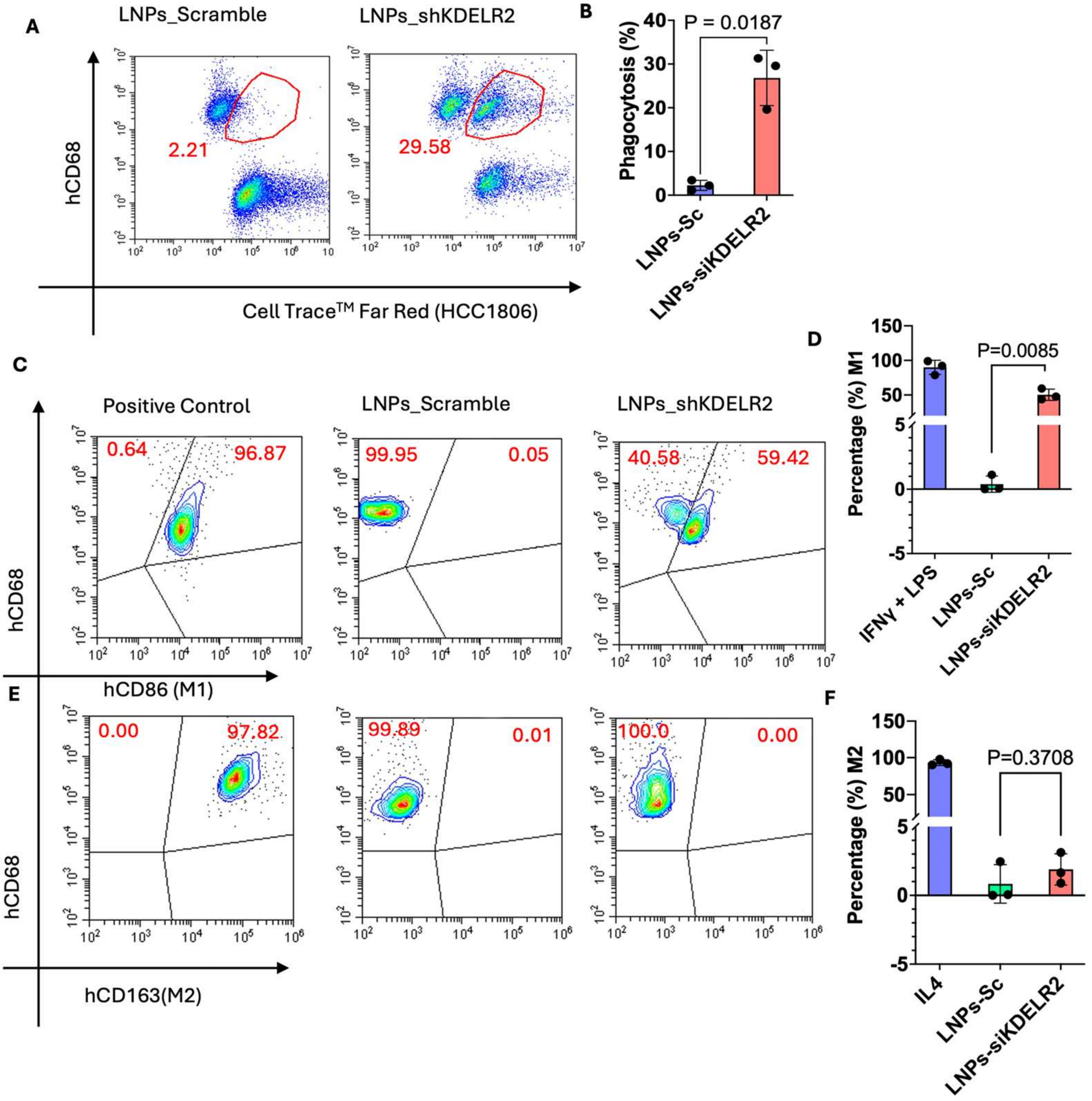

Figure S7: Suppression of KDELR2 in HCC1806 cells promotes opsonization and M1 polarization of human macrophages. A. HCC1806 human breast cancer cells were with LNPs loaded with scramble or siRNA to KDELR2. 24 h later cells were labeled with far red CellTrace and incubated with M1 polarized human primary macrophages labeled with FITC-conjugated anti-human CD68. Shown is one of three replicates performed with the same donor cells and the quantification of the double positive population (**B**). **C.** Naïve human macrophages were incubated for 48 h with conditioned media of HCC1806 treated with LNPs loaded with siKDELR2 or a scramble control and analyzed for the M1 marker CD86. IFNγ/LPS was used as a positive control for M1 polarization. **D.** Quantification of three independent experiments performed with the same donor cells (Mean ± SD). **E.** Same as in C. Analysis was performed for the M2 marker CD163. **F.** Quantification of three independent experiments performed with the same donor cells (Mean ± SD). N = 3 for each experiment. Statistical significance was determined using Welch's *t*-test; $p < 0.05$ was considered significant

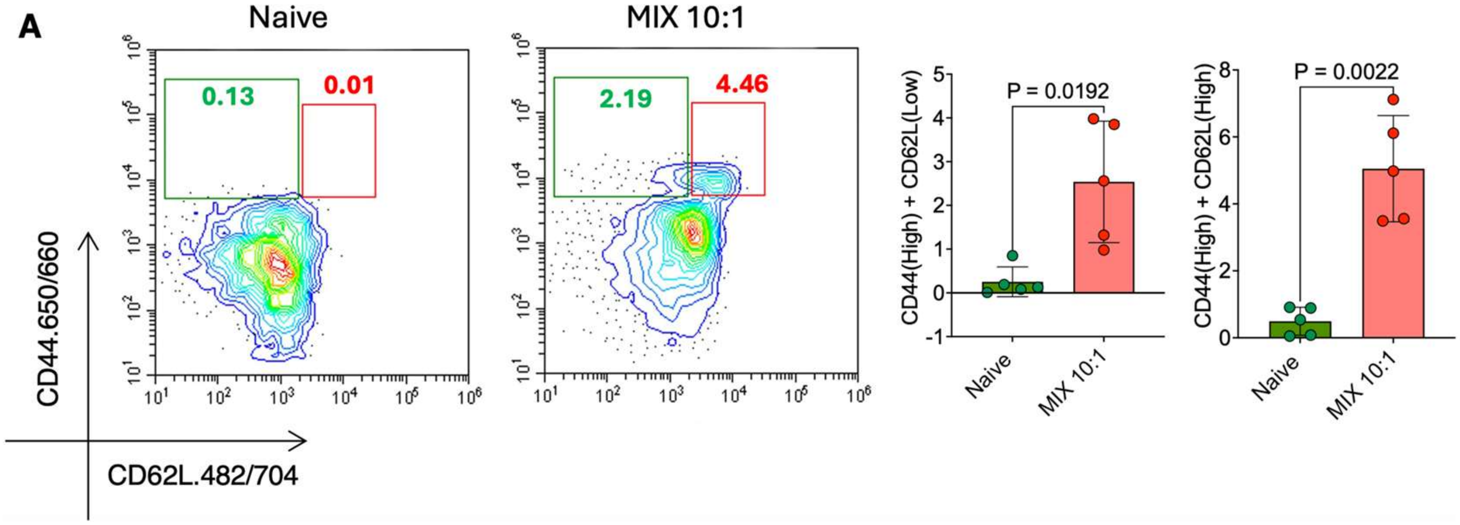

Figure S8. KDELR2-driven tumor regression induces systemic memory T cell responses. C57BL/6 mice were challenged with MIX 10:1 tumors and allowed to develop measurable tumors prior to induction of KDELR2 silencing via doxycycline (DOX) administration. Mice were maintained on DOX until complete tumor regression. Two weeks following regression, spleens were harvested, red blood cells were lysed, and T cells were analyzed by flow cytometry for CD44 and CD62L expression. Naïve mice maintained in parallel under identical dietary conditions served as controls.

A. Representative flow cytometry plots show CD44 and CD62L expression on T cells. **B.** Quantification demonstrates a significant increase in CD44^high CD62L^low effector/memory T cell populations in mice that underwent tumor regression compared to naïve controls (N = 5 per group, mean ± SD). Statistical significance was determined using Welch's unpaired t-test, with $p < 0.05$ considered significant.

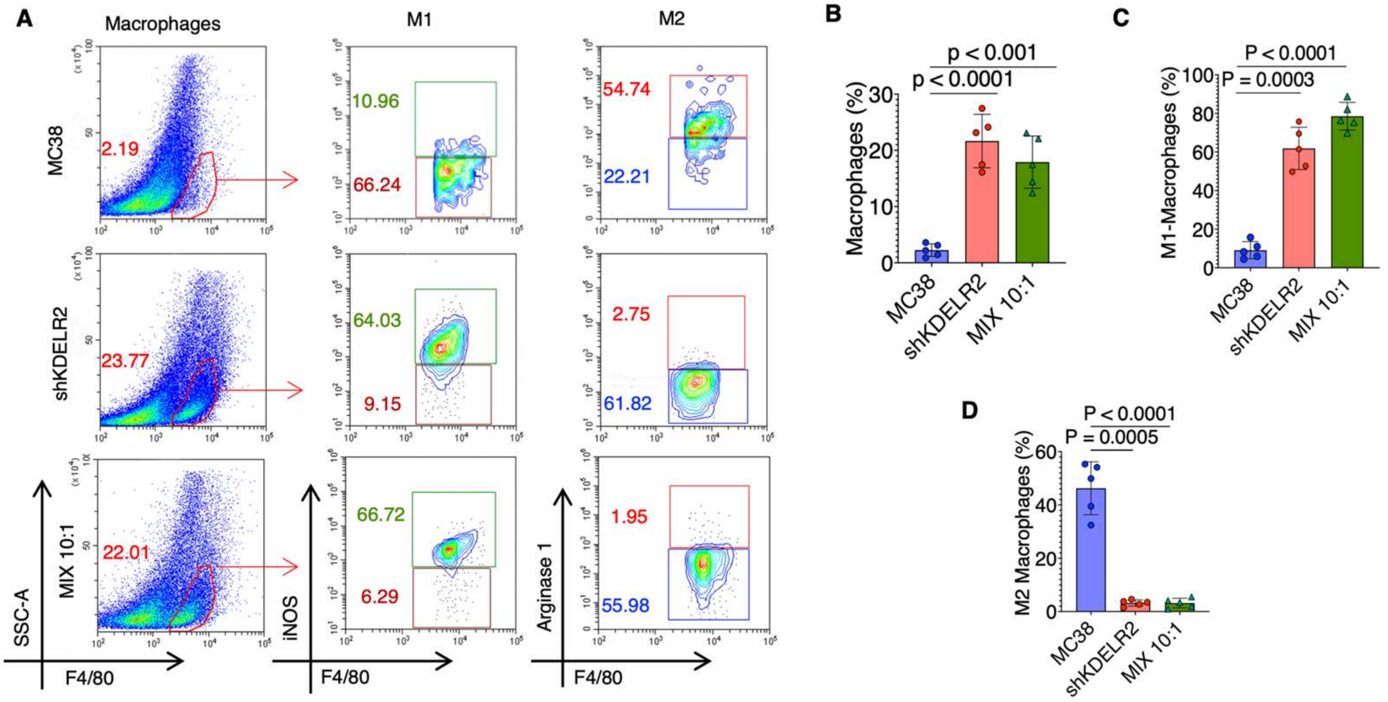

Figure S9: Characterization of infiltrated macrophages into MC38 tumors after regression of primary tumors. A. Representative flow cytometry analyses for total macrophages and their polarization in MC38-ffLuc tumors, when grown two weeks after the regression of Tet-On shKDELR2 tumors. Average ± SD (N = 5) of five different tumors is shown for total macrophages (**B**), M1 (**C**) and M2 (**D**). Statistical significance was determined using Welch's unpaired *t*-test; $p < 0.05$ was considered significant

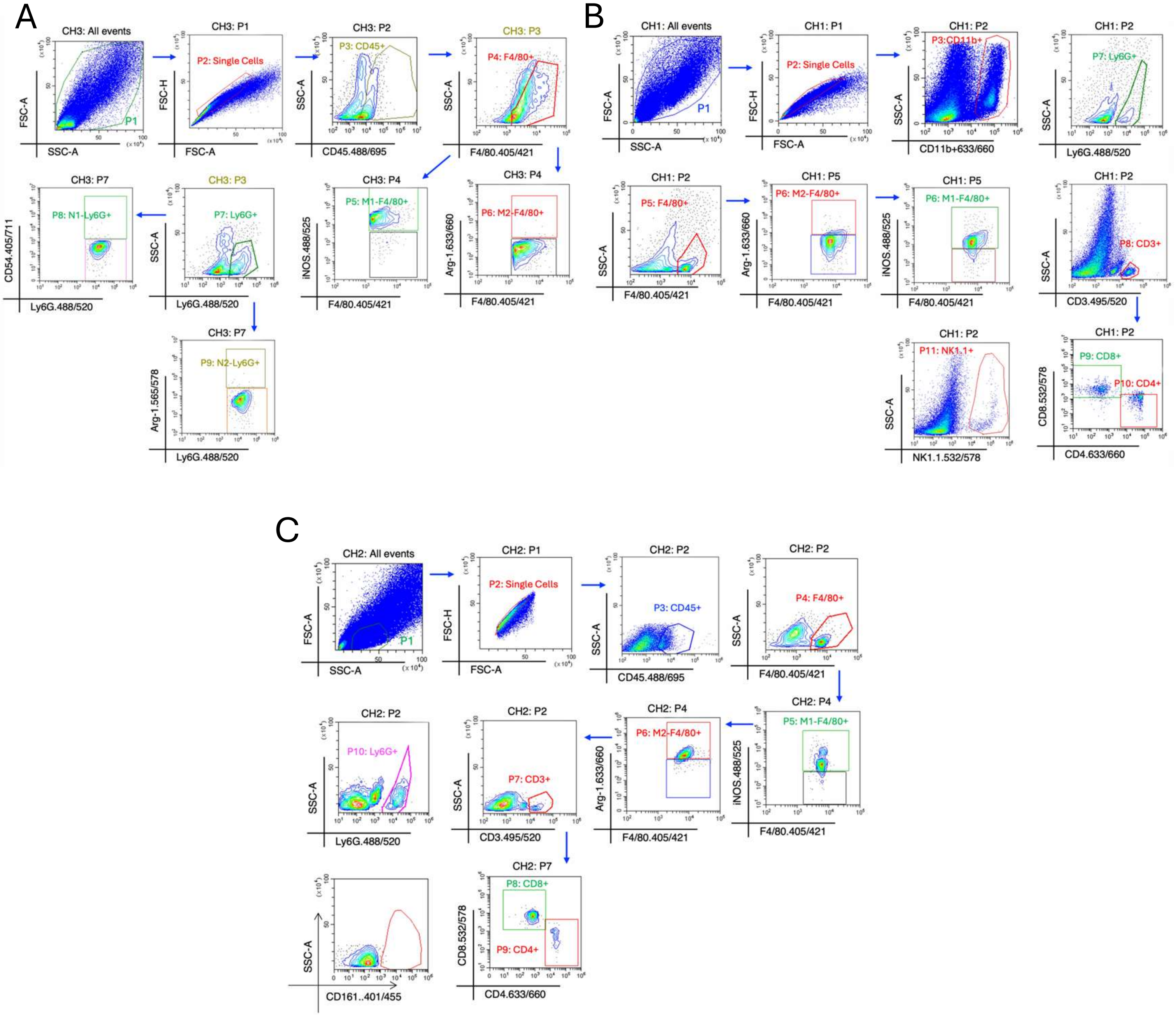

Figure S10: Multiparametric flow cytometry gating strategy for comprehensive characterization of innate and adaptive immune populations in the tumor microenvironment in xenograft and orthotopic tumor models. Flow cytometry gating was employed to characterize immune cell populations within the TME of both xenograft and orthotopic tumor models using different strategies. The strategy involved sequential gating on live, singlet cells after SSC-A, which was used as initial population separation: **A.** selection of total CD45^{+} immune cells (Figure S5, S6), **B.** using tumor immune microenvironment selection (Figure 2, 4, 7), **C.** using selective gating strategies for lymphocyte/monocytes (Figure 5, 6). From this population, immune subsets were identified, including enriched-macrophages (F4/80^{+}), neutrophils (Ly6G^{+}), and T cells (CD3^{+}), further with CD4+/CD8+ cells. Macrophage polarization was assessed using iNOS (M1) and Arg-1 (M2) markers. Further, after the total CD45+ immune cells selection in S10(A), neutrophil subsets were characterized based on CD54 and Arg-1 expression to distinguish non-suppressive (N0/N1-like) and suppressive (N2-like) phenotypes. NK cells were identified as NK1.1^{+}/CD161. Representative plots from independent experiments are shown. All gates were defined using fluorescence minus one (FMO) controls, isotype controls, and Fc-blocking (CD16/CD32) to ensure specificity and minimize nonspecific binding.

Note: Some gating strategy panels may overlap with main figures because selected representative plots were intentionally included for illustrative purposes.

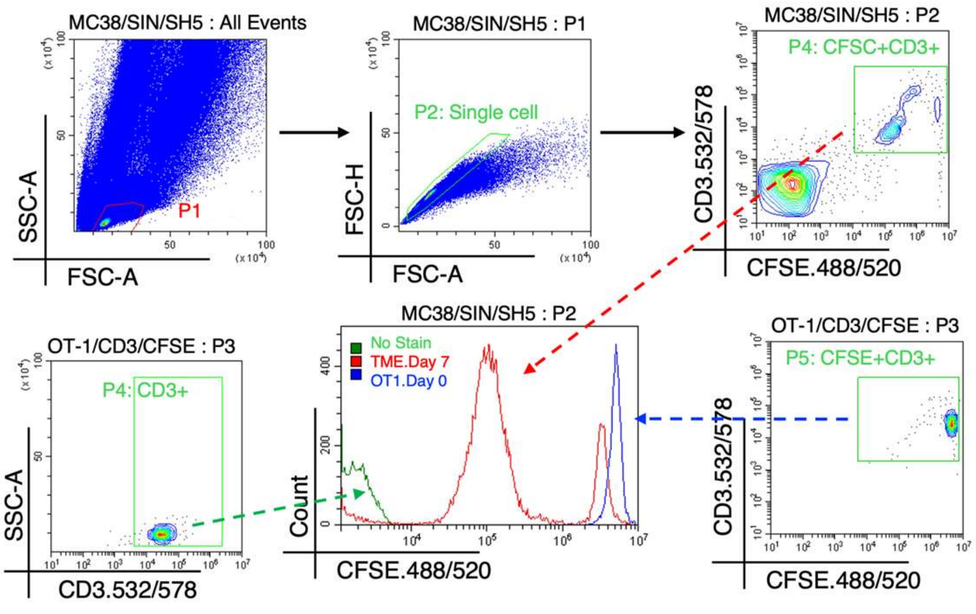

Figure S11: Gating Strategy for Analyzing T-Cell Priming in the Tumor Microenvironment (TME) Following Intravenous Implantation of OT-I T Cells. Flow cytometric gating was performed to evaluate T-cell priming within the TME after intravenous administration of OT-I T cells (Figure7).

Note: Some gating strategy panels may overlap with main figures because selected representative plots were intentionally included for illustrative purposes.
